# CellTracer: a comprehensive database to dissect the causative multilevel interplay contributing to cell development trajectories

**DOI:** 10.1093/nar/gkac892

**Published:** 2022-10-16

**Authors:** Qiuyan Guo, Peng Wang, Qian Liu, Yangyang Hao, Yue Gao, Yue Qi, Rongji Xu, Hongyan Chen, Mengyu Xin, Xiaoting Wu, Rui Sun, Hui Zhi, Yunpeng Zhang, Shangwei Ning, Xia Li

**Affiliations:** Department of Gynecology of the First Affiliated Hospital, College of Bioinformatics Science and Technology, Harbin Medical University, Harbin 150081, China; Department of Gynecology of the First Affiliated Hospital, College of Bioinformatics Science and Technology, Harbin Medical University, Harbin 150081, China; Department of Gynecology of the First Affiliated Hospital, College of Bioinformatics Science and Technology, Harbin Medical University, Harbin 150081, China; Department of Gynecology of the First Affiliated Hospital, College of Bioinformatics Science and Technology, Harbin Medical University, Harbin 150081, China; Department of Gynecology of the First Affiliated Hospital, College of Bioinformatics Science and Technology, Harbin Medical University, Harbin 150081, China; Department of Gynecology of the First Affiliated Hospital, College of Bioinformatics Science and Technology, Harbin Medical University, Harbin 150081, China; Department of Gynecology of the First Affiliated Hospital, College of Bioinformatics Science and Technology, Harbin Medical University, Harbin 150081, China; Department of Gynecology of the First Affiliated Hospital, College of Bioinformatics Science and Technology, Harbin Medical University, Harbin 150081, China; Department of Gynecology of the First Affiliated Hospital, College of Bioinformatics Science and Technology, Harbin Medical University, Harbin 150081, China; Department of Gynecology of the First Affiliated Hospital, College of Bioinformatics Science and Technology, Harbin Medical University, Harbin 150081, China; Department of Gynecology of the First Affiliated Hospital, College of Bioinformatics Science and Technology, Harbin Medical University, Harbin 150081, China; Department of Gynecology of the First Affiliated Hospital, College of Bioinformatics Science and Technology, Harbin Medical University, Harbin 150081, China; Department of Gynecology of the First Affiliated Hospital, College of Bioinformatics Science and Technology, Harbin Medical University, Harbin 150081, China; Department of Gynecology of the First Affiliated Hospital, College of Bioinformatics Science and Technology, Harbin Medical University, Harbin 150081, China; Department of Gynecology of the First Affiliated Hospital, College of Bioinformatics Science and Technology, Harbin Medical University, Harbin 150081, China

## Abstract

During the complex process of tumour development, the unique destiny of cells is driven by the fine-tuning of multilevel features such as gene expression, network regulation and pathway activation. The dynamic formation of the tumour microenvironment influences the therapeutic response and clinical outcome. Thus, characterizing the developmental landscape and identifying driver features at multiple levels will help us understand the pathological development of disease in individual cell populations and further contribute to precision medicine. Here, we describe a database, CellTracer (http://bio-bigdata.hrbmu.edu.cn/CellTracer), which aims to dissect the causative multilevel interplay contributing to cell development trajectories. CellTracer consists of the gene expression profiles of 1 941 552 cells from 222 single-cell datasets and provides the development trajectories of different cell populations exhibiting diverse behaviours. By using CellTracer, users can explore the significant alterations in molecular events and causative multilevel crosstalk among genes, biological contexts, cell characteristics and clinical treatments along distinct cell development trajectories. CellTracer also provides 12 flexible tools to retrieve and analyse gene expression, cell cluster distribution, cell development trajectories, cell-state variations and their relationship under different conditions. Collectively, CellTracer will provide comprehensive insights for investigating the causative multilevel interplay contributing to cell development trajectories and serve as a foundational resource for biomarker discovery and therapeutic exploration within the tumour microenvironment.

## INTRODUCTION

Cancer cells within the same tumour exhibit phenotypic and functional heterogeneity due to genetic variants and constant changes in the microenvironment (1). The dynamic development of tumour heterogeneity not only improves the ability of a tumour to adapt to ever-changing constraints but also influences patient prognosis, therapy response, and clinical outcome (2). Single-cell RNA sequencing (scRNA-seq) technology (3) can explore tissue heterogeneity by isolating distinct cell populations and greatly expanding our knowledge of complex microbial ecosystems (4). Currently, many single-cell sequencing-dependent methods have been established to measure various molecular layers, including proteomics (5), epigenomics (6), and transcriptomics (7). However, the dynamic changes in cell development processes, such as cell-state transition and pathway activation, are not always well described by single-cell assays and traditional methods. For example, T(H) cell differentiation is accompanied by dynamic changes in histone acetylation of cytokine genes (8). NK cells gradually develop from haematopoietic stem cells (HSCs) through common lymphoid progenitors (CLPs) and NK-cell precursors (NKPs) and then migrate to peripheral blood (cNK cells) or tissue (trNK cells) (9). As an important development in scRNA-seq-dependent technology, cell trajectory analysis can verify known cell differentiation relationships at single-cell resolution (10), reveal cell developmental lineages (11), explore the dynamic changes in tumour microenvironments (12), and determine causative cell subsets in and the driver genes of disease development (13).

To explore disease pathology at the individual level, several works have been carried out by characterizing the crosstalk between different molecular layers based on bulk expression data (14–16). With the rapid development of scRNA-seq technology, some powerful databases, such as DISCO (17), CancerSCEM (18), LnCeCell (19) and Cell Blast (20), have been developed. DISCO focuses on the deep integration of human scRNA-seq data. CancerSCEM provides a scRNA-seq dataset of 20 human cancers and performs gene expression comparisons between different scRNA-seq and TCGA bulk RNA-seq datasets. LnCeCell is a database focusing on the identification of ceRNA regulatory networks at single-cell resolution. Cell Blast is an effective cell-querying method built on a neural network model. Furthermore, some single-cell analysis pipelines have been developed to study cell development trajectories. For example, STREAM can disentangle and visualize complex branching trajectories from both single-cell transcriptomic and epigenomic data (21). scSTEM provides a method for clustering the dynamic profiles of genes in trajectories inferred from the pseudotime ordering of scRNA-seq data (22). The emergence of these valuable works can make it easier to explore disease pathology at single-cell resolution and provides novel insights into tumour microenvironments. However, a database dedicated to characterizing the complex cell development landscape and identifying driver features at multiple levels is still lacking. Furthermore, a fast and flexible online tool focusing on the visualization of multilevel crosstalk between different contexts along distinct cell trajectories remains to be developed.

To fill these gaps, we developed CellTracer, a comprehensive database to dissect the causative multilevel interplay contributing to cell development trajectories. CellTracer collects the scRNA-seq profiles and cell-state annotations of 1 941 552 cells from 222 datasets, including (i) 118 datasets of 42 diseases with different clinical treatments, such as chemotherapy, immunotherapy, or other targeted therapy methods; (ii) 104 normal datasets from 80 healthy organ/tissue types; and (iii) >10 000 biological functional contexts, such as Gene Ontologies, pathways, and hallmarks; (iv) clusters of distinct cell populations exhibiting diverse behaviours, such as angiogenesis, apoptosis, cell cycle, invasion, proliferation, and stemness and (v) customized dynamic heatmaps illustrating detailed gene expression across 117 cell types and distinct cell development trajectories. To trace the development trajectories of cells with different fates and identify the intrinsic development-dependent features, CellTracer also provides a set of interactive tools that facilitate retrieval, visualization and analysis. We expect the CellTracer database to serve as an important resource for investigating causative multilevel interplay contributing to cell development trajectories, aiding in the understanding of regulatory mechanisms behind complex cell development processes.

## MATERIALS AND METHODS

### Data collection and processing

In CellTracer, scRNA-seq expression profiles and metadata information (including sample ID, organ/tissue origin, clinical treatment, biosample groups, etc.) were collected from Gene Expression Omnibus (GEO) (23), ArrayExpress (24) and TISCH (25). Gene annotation files were collected from GENCODE (release 41, GRCh38) (26) to identify different types of genes, including protein-coding genes, long noncoding RNAs (lncRNAs), pseudogenes, etc. Datasets with more than 100 cells were retained in CellTracer. After raw data normalization and quality control, CellTracer documented 1 941 552 cells from 222 datasets, including 118 datasets of 42 diseases and 104 datasets of 80 healthy organs/tissues. In cell-type identification, CellTracer used original cell-type annotation (if provided by the original data source) or performed the CELLiD method to perform annotation analysis (17). To provide comprehensive annotations of diverse cell types, we integrated cell markers from both DISCO (17) and CellMarker (27) as cell-type annotation references. To dissect the functional activation status and state transition of individual cell populations, CellTracer collected the gene sets of more than 10 000 biological functional contexts, including Gene Ontologies (28), biological pathways (29), cancer hallmarks (30) and cell states (31). The gene set variation analysis (GSVA) method (32) was used to evaluate the cellular functional activation status and states in each dataset. To identify clusters of distinct cell populations, the tSNE and UMAP clustering algorithms were implemented in the Seurat R package (v4.0.2) (33). The Monocle 2 package (v2.18.0) (34) was used to calculate pseudotime and states and further construct cell development trajectories by applying the DDRTree reduction method. For each dataset, CellTracer provided trajectory analyses for different cell types, such as malignant cells, immune cells, and stromal cells. Furthermore, the trajectory analysis results of Monocle 3 (v1.2.9) (35) were also included in CellTracer. Users can choose different methods (Monocle 2 and Monocle 3) to perform trajectory analyses. Detailed information on data collection and processing is provided in the Supplementary Methods.

### Database construction

CellTracer is freely available at http://bio-bigdata.hrbmu.edu.cn/CellTracer/. The online web server of CellTracer was constructed by Java Server Pages language and deployed on Tomcat software (v6). All datasets of CellTracer were documented and managed based on the MySQL data source server (v5.5). Several Java script packages, including jQuery (v1.11.3), Datatable (v1.10.10) and ECharts (v4.0), were implemented for result data creation and multilevel data cross-talk visualization. All data processing and statistical analyses were performed using R software (v4.2.1). Currently, the CellTracer website can be well supported by state-of-the-art web browsers, such as Safari, Google Chrome, Microsoft Edge and Firefox.

## RESULTS

### Data collection and content of CellTracer

**Figure 1.**
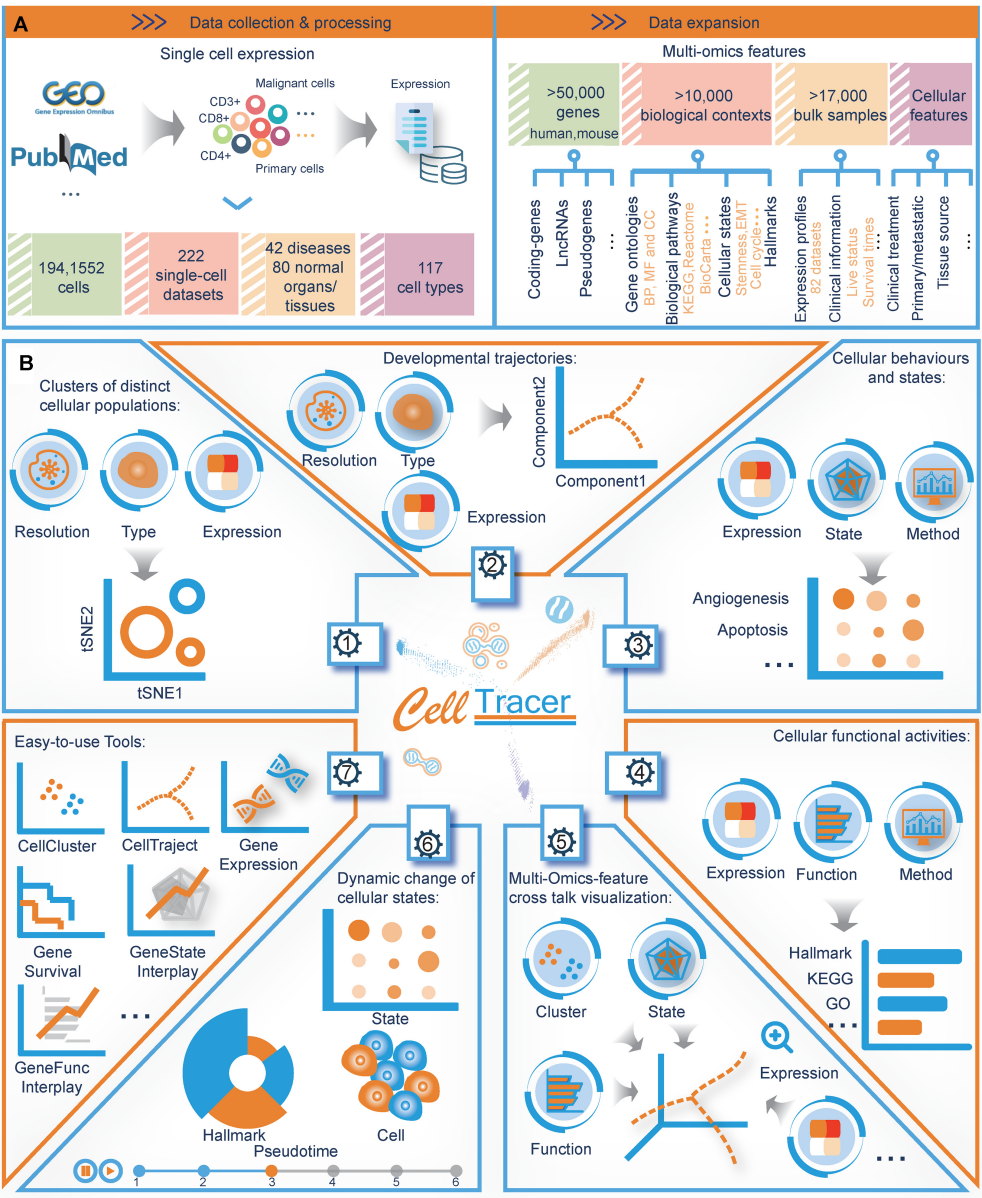
Overview of data content and functions of CellTracer. (**A**) The top panels demonstrate the dataset content and expansion of multilevel features. (**B**) The middle and bottom panels demonstrate the functional frameworks to retrieve, analyse and visualize the scRNA-seq data.

The current version of CellTracer collected the scRNA-seq profiles of 1 941 552 cells from 222 single-cell datasets (Figure 1A). There were 118 datasets curated from 42 common human diseases, such as cancer and coronavirus disease 2019 (COVID-19), and 104 datasets curated from 80 healthy organs/tissues (Supplementary Figure S1A–D). For each dataset, CellTracer collected clinical information such as organ/tissue origin, clinical treatment and biosample groups to provide a comprehensive description. After raw data normalization and quality control, the average number of cells in each dataset was 8745, with melanoma (GSE157743) having the smallest number of single cells (*n* = 102) and COVID-19 (GSE167118) having the largest number (*n* = 54 094). In each single cell, CellTracer performed a cell-type annotation pipeline and identified expression profiles for different gene types, including coding genes, lncRNAs, pseudogenes, etc. Finally, a total of 51 213 unique genes and 117 cell types were covered in CellTracer. We observed that most genes could be detected in <20 datasets, indicating specific expression patterns and functions at single-cell resolution (Supplementary Figure S2). On the other hand, 3042 genes were expressed in >200 datasets. Most of these genes were housekeeping genes (36), indicating their constant expression levels in all conditions and basic cell maintenance functions. The 117 cell types were diversely distributed in both disease and normal datasets (Supplementary Figures S3 and S4). For example, malignant cells were found in most human cancer datasets, whereas CD4+ T cells were frequently found in COVID-19 and bacterial pneumonia datasets.

To characterize the developmental landscape and identify driver features at the single-cell level, CellTracer identified clusters of distinct cell populations and constructed development trajectories for each dataset (Figure 1B). In addition to gene expression, CellTracer also collected multilevel features to study cellular behaviours and fates. These features consisted of (i) the functional annotations of gene ontology (GO) terms, (ii) biological pathways, (iii) hallmarks representing specific well-defined biological processes, (iv) the functional states of cancer cells and (v) cell characteristics such as cell types, development states and pseudotime. By using CellTracer, users can explore causative features and their interplay within distinct cell clusters and development trajectories. As an important supplement, 82 bulk expression profiles and individual clinical information for 17 777 cancer samples were collected into CellTracer for prognostic analysis (Supplementary Figure S5).

### Features and utilities of CellTracer

#### Flexible ways to query and access the dataset

**Figure 2.**
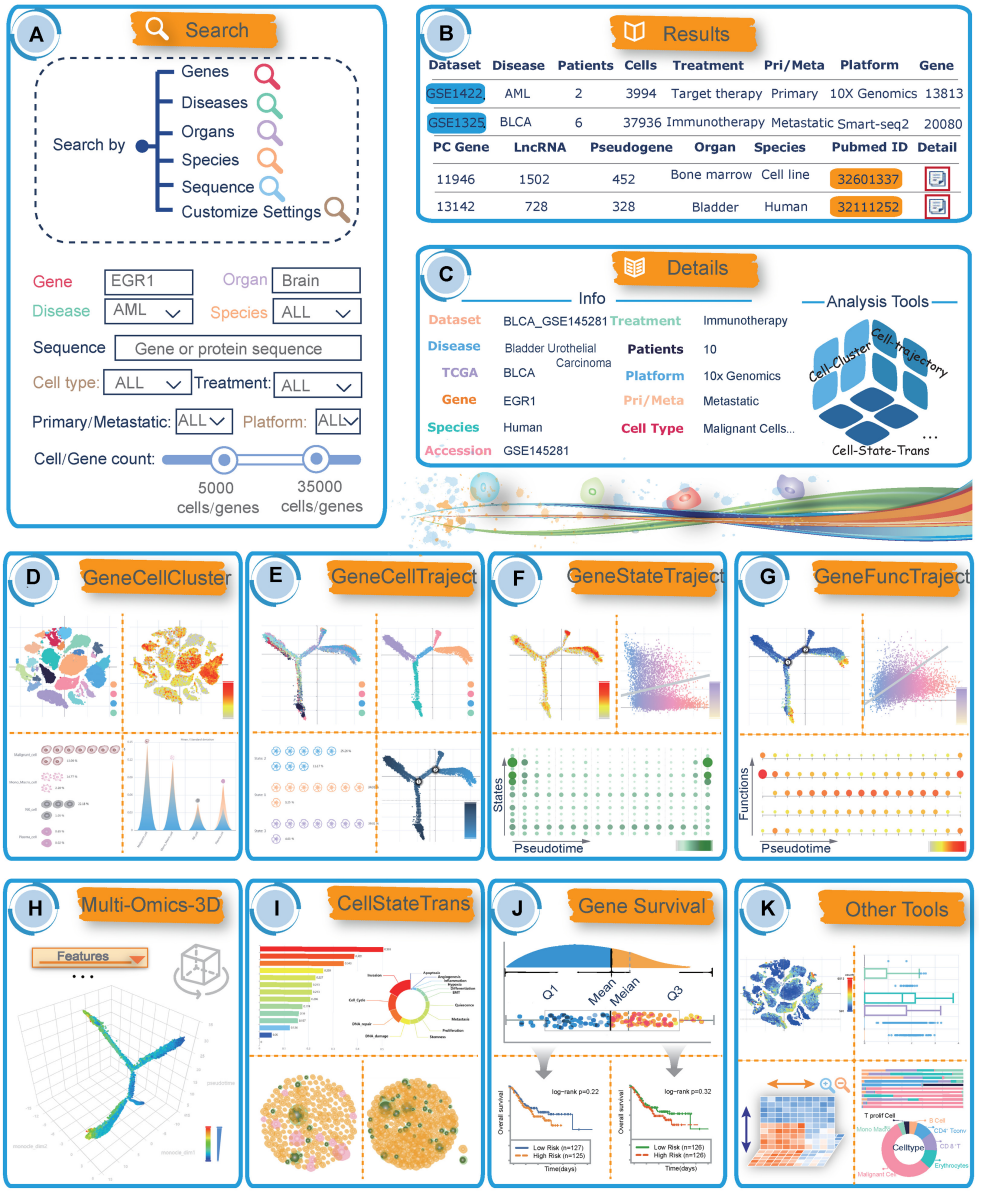
Features and utilities of CellTracer. (**A**) The quick and advanced search interfaces of CellTracer. (**B**) The result data table of CellTracer. (**C**) Detailed descriptions and multifeature interfaces for data analysis. (**D–K**) A panel of user-friendly analysis tools in CellTracer.

CellTracer provides easy-to-use interfaces for data accession and visualization (Figure 2A). On the home page, a ‘QUICK SEARCH’ interface is available to directly investigate data or perform analyses (Supplementary Figure S6). Users can flexibly explore the CellTracer database by inputting different types of keywords, including gene names/IDs, diseases, organs/tissues, primary/metastatic sites, species, platforms, cell types, treatments, gene/protein sequences and dataset accession IDs. CellTracer also provides an advanced search interface for users to perform exploration based on different query criteria (Supplementary Figure S7). As a result, CellTracer returns a data table in which each row provides a description of related datasets (Figure 2B) and an illustration of the composition of cell types and the distribution of gene expression (Supplementary Figure S8). For an input gene, CellTracer also provides an interactive heatmap illustrating the detailed gene expression across different datasets and cell types. Users can directly reorder the rows and columns of the heatmap by setting different ranking parameters (such as gene expressions, gene counts, and clusters) (Supplementary Figure S9). For each dataset, CellTracer provides detailed descriptions and multifeature interfaces for further analysis (Figure 2C).

#### A panel of user-friendly analysis tools

In addition to data query and accession, CellTracer also provides a panel of user-friendly tools (including six comprehensive tools and six mini tools) for the retrieval and analysis of data. Each comprehensive tool implements a proposed analysis pipeline by integrating different methodologies and multilevel data. For example, the GeneCellCluster tool explores the gene expression and cellular distribution of different clusters, cell types, states, primary/metastatic sites, etc. (Figure 2D and Supplementary Figure S10). The GeneCellTraject tool constructs the development trajectories inferred from cellular pseudotime and illustrates the detailed feature distribution of cell subpopulations along distinct lineages (Figure 2E and Supplementary Figure S11). The GeneStateTraject and GeneFuncTraject tools evaluate functional activities and states at the single-cell level and perform correlation analysis between gene expression and functional contexts to identify intrinsic branch-dependent features (Figure 2F, G and Supplementary Figures S12 and S13). At the bottom of these web pages, a trajectory plot illustrating different cell branches has also been provided as a reference. Users can better know which branch the gene or state events should be assigned to. The Multi-Omics-3D tool performs multilevel interplay analysis, which contributes to cell development trajectories and cell fates in a three-dimensional view (Figure 2H and Supplementary Figure S14). The CellStateTrans tool illustrates the dynamic changes in cell states/behaviours along specific development branches (Figure 2I and Supplementary Figure S15). CellTracer develops several easy-to-use mini tools to perform brief or simple analysis (Figure 2J, K). For example, the GeneSurvival tool performs Cox analysis and creates Kaplan−Meier curves for genes based on >17 000 cancer samples from 82 bulk sequencing datasets.

### Example application of CellTracer

#### Construction of a development trajectory within tumour-infiltrating lymphocytes

**Figure 3.**
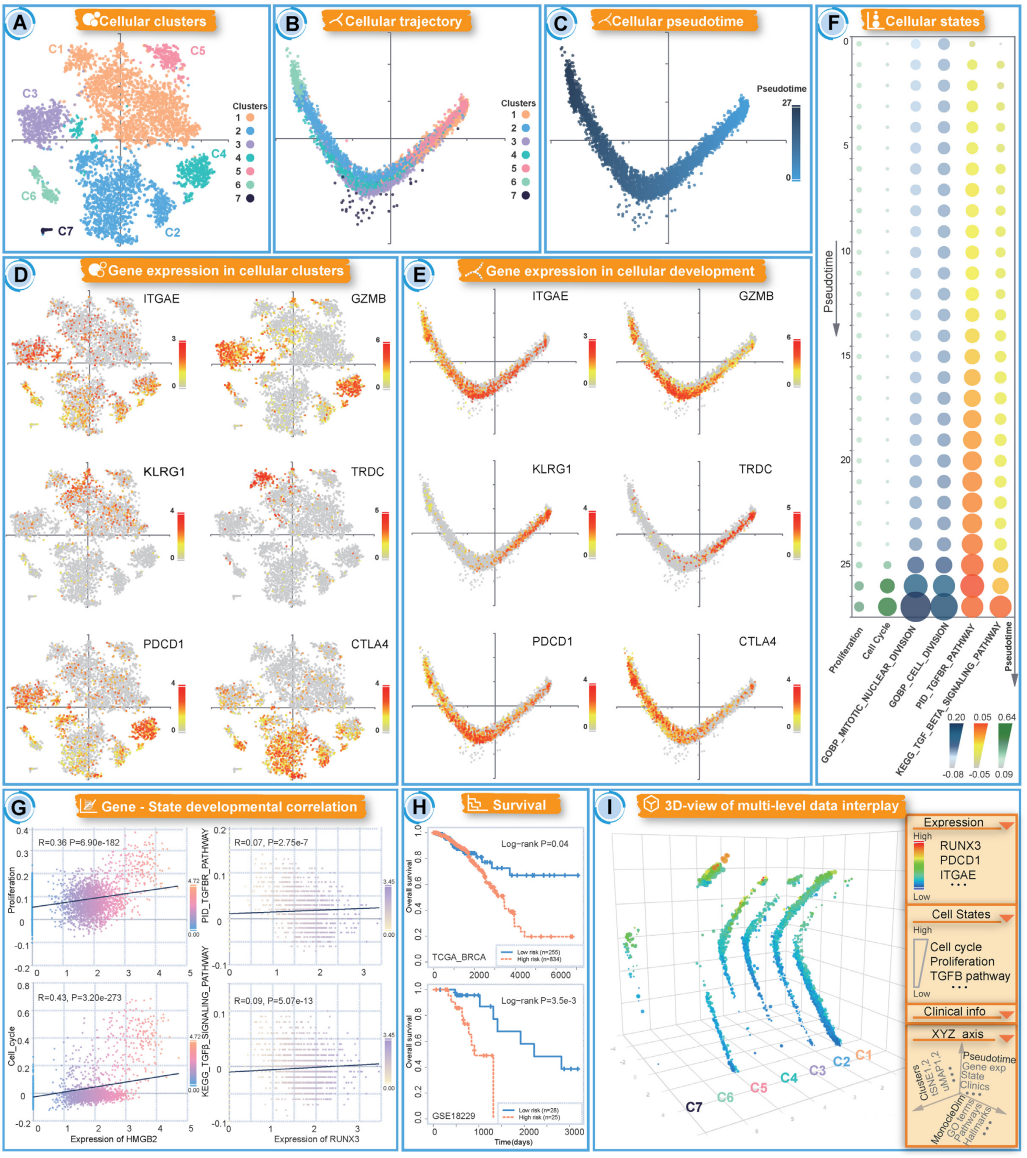
Examples of CellTracer workflow and application in dissecting tumour-infiltrating microenvironments. (**A**) Distinct T-cell clusters revealed by CellTracer. The dim reduction was performed by the TSNE method. (**B, C**) The cell development trajectory with different clusters and pseudotime constructed by CellTracer. The dim reduction was performed by Monocle. (**D, E**) Marker gene expression across cell clusters and lineages. (**F**) The dynamic changes in functional activities and cell states based on different contexts. (**G**) The multilevel interplay contributing to the development of the T-cell-state transition. (**H**) Survival analysis of the TRM marker gene ITGAE in bulk expression data. (**I**) Combination and illustration of multilevel feature interplay.

To demonstrate the potential application of CellTracer in characterizing the developmental landscape and identifying driver features at multiple levels, we performed an analysis on a scRNA-seq dataset of breast cancer T cells (GSE110686). Cell clusters and development trajectories were constructed and visualized through the GeneCellCluster and GeneCellTraject tools of CellTracer (Figure 3A–C). We found that T cells were distributed into seven unique clusters with different cellular pseudotimes along the development trajectory, indicating complex tumour-infiltrating microenvironments with diverse T-cell subpopulations and cell states. To further explore the diverse cellular compositions, we used the GeneExpression tool of CellTracer to visualize marker gene expression across cell clusters and lineages (Figure 3D, E). Four clusters (C2, C3, C4 and C6) had high expression of marker genes (ITGAE and GZMB), suggestive of a tissue-resident memory T (TRM) cell phenotype (37,38). Cluster C1 had high expression of marker genes (KLRG1 and TRDC) for T effector memory (TEM) cells and a small subset of γδ T cells (39,40). The composition of TRM and TEM cells revealed diverse T-cell subpopulations with different immune states (Supplementary Figure S16). TRM cells exhibit tissue residency and provide rapid and superior control of localized infections (41), whereas TEM cells meditate protective memory by migrating to inflamed peripheral tissues (42). We observed that TRM and TEM cells were localized at opposite ends of the development trajectory (Figure 3B, E), indicating the distinct gene expression profiles and dynamic state transition of these cells (43). High expression of several immune checkpoints, such as PDCD1 and CTLA4, was observed in TRM cells, which is consistent with previous studies (43,44) showing that TRM cells expressed high levels of immune checkpoint molecules (Figure 3D-E).

#### Dissection of functional heterogeneity along developmental lineages

To dissect the phenotypic and functional heterogeneity of tumour-infiltrating lymphocytes, we used the GeneStateTraject and GeneFuncTraject tools of CellTracer to evaluate the cell-state transitions based on different functional contexts (Figure 3F). A previous study reported a subpopulation of TRM cells that displayed mitotic features with proliferation activity distributed on the end of the pseudotime path (43). This cellular heterogeneity was also captured by the CellTracer analysis pipeline. The C6 subpopulation was found to be localized at the end of the cell trajectory (with high pseudotime) with high expression of the proliferation gene HMGB2 (Figure 3A–C, Supplementary Figure S17). Increased cell activities of proliferation, cell cycle and division were also observed in the C6 subpopulation with relatively higher pseudotime (Figure 3F). Based on the results of the CellTracer-GeneStateTraject tool, HMGB2 expression was positively correlated with cell proliferation and the cell cycle score (Figure 3G). These observations revealed the heterogeneity of TRM cells by characterizing a subset of cell populations undergoing proliferation and division states.

#### Identification of driver features contributing to cell-state transition

To further identify potential driver features and multilevel interplay contributing to the development of the T-cell-state transition, we used the GeneFuncTraject tool of CellTracer to evaluate transforming growth factor β-responsive (TGF-β) pathway activity at single-cell resolution (Figure 3F). The TGF-β pathway is required for the formation and maintenance of tissue residency of TRM cells (38). Increased TGF-β activity was observed within the cell development trajectory (from lower to higher pseudotime), indicating its contribution to TRM cell formation (Figure 3F). Furthermore, we focused on the transcription factors that were highly expressed in TRM cell clusters to identify essential genes required for the formation of TRM cells. We found that the RUNX3 gene was highly expressed in the C4 and C6 clusters and moderately expressed in the C2 and C3 clusters (Supplementary Figure S18). In the cell trajectory, the C2 and C3 clusters (with lower pseudotime) were localized adjacent to the C4 and C6 clusters (with higher pseudotime), indicating the developmental process of TRM cell formation (Figure 3B). A recent study demonstrated that RUNX3 is a critical regulator of CD8 + T-cell tissue residency, while cells with RUNX3 deficiency lack the TGF-β transcriptional network that underpins tissue residency (41). We used the GeneFuncTraject tool to explore the interplay between RUNX3 expression and TGF-β pathway activity in T cells, and we found a positive correlation between these molecular and functional features (Figure 3G), indicating that RUNX3 is a potential driver gene in TRM cell development. Previous studies revealed that the TRM gene signature identified from the scRNA-seq profile was significantly associated with patient survival (43,45). We used the GeneSurvival tool of CellTracer to perform survival analysis of the TRM marker gene ITGAE (Figure 3D) across a panel of 16 breast cancer bulk datasets, and we found that ITGAE was a prognostic factor in the TCGA and GSE18229 datasets (Figure 3H and Supplementary Figure S19).

#### Combination and illustration of multilevel feature interplay

In the above, we performed an analysis of cell clusters, cell trajectories, marker gene expression and cell states based on different CellTracer tools and purposes. These multilevel features and their crosstalk can be combined and visualized by the Multi-Omics-3D tool of CellTracer. Users can simultaneously map these multilevel features to the *x*-, *y*-, *z*-axes and assign node colours and symbol sizes. As an example of the Multi-Omics-3D tool in Figure 3I, distinct T-cell clusters can be directly visualized with different cell development trajectories. Based on this construction, the exploration of the crosstalk among molecular features (such as the TRM cell marker ITGAE, immune checkpoint PDCD1 and TGF-β pathway driver gene RUNX3), functional and state features (such as cell cycle, proliferation and TGF-β pathway) and clinical features (such as stage, source and treatments) is easy and flexible. More usage and examples of the Multi-Omics-3D tool are illustrated in Supplementary Figure S20.

## DISCUSSION

ScRNA-seq technologies have greatly expanded our knowledge of complex tumour microenvironments and heterogeneity by evaluating gene expression at the single-cell level. The development of tumour heterogeneity is a dynamic process with continuous gene expression variation and cell-state or behaviour transitions. Although some valuable works have focused on capturing cell behaviours and made it possibly easier to understand how diverse cells contribute to overall tissue behaviour, a comprehensive database dedicated to characterizing the complex cell development landscape and identifying driver features at multiple levels is still lacking. To meet these needs, we developed CellTracer, a comprehensive database to dissect the causative multilevel interplay contributing to cell development trajectories. CellTracer curated the scRNA-seq expression profiles and state annotations of 1,941,552 cells from 222 single-cell datasets. For each dataset, CellTracer identified clusters of distinct cell populations and constructed their development trajectories to detect genetic variants and constant changes in cell fates. In CellTracer, both the Monocle 2 and Monocle 3 analysis pipelines were used. The most important difference between Monocles 2 and 3 is that the DDRTree-based method assumes that trajectories are connected into a single tree-like structure. In Monocle3, multiple, disjointed graphs can be learned. CellTracer also provides a set of interactive tools that facilitate the retrieval, visualization and analysis of data. Through CellTracer, users can explore the significant alterations in molecular events and the causative multilevel crosstalk among genes, biological contexts, cell characteristics and clinical treatments along distinct cell development trajectories. The combination of these features will help us understand the pathological development of disease in individual cell populations and further contribute to precision medicine. With the fast-growing number of scRNA-seq datasets, an increasing number of disease types will be characterized by scRNA-seq technology. To provide up-to-date information on diseases, we will continuously update CellTracer by integrating more datasets and functional tools in the future.

## DATA AVAILABILITY

All the data used in the analysis can be obtained at http://bio-bigdata.hrbmu.edu.cn/CellTracer.

## Supplementary Material

gkac892_Supplemental_File
