# Supplementary material for: CellTracer: a comprehensive database to dissect the causative multilevel interplay contributing to cell development trajectories
Source: Nucleic Acids Res. 2022 Oct 16;51(D1):D861–9. doi: 10.1093/nar/gkac892 (PMC9825461; doi:10.1093/nar/gkac892)
Supplement: gkac892_Supplemental_File [file gkac892_supplemental_file.pdf]

Supplementary Methods

Data collection and pre-processing

We systematically collected scRNA-seq datasets of human disease and healthy organ/tissue from Gene Expression Omnibus (GEO, https://www.ncbi.nlm.nih.gov/geo/) (1), ArrayExpress (https://www.ebi.ac.uk/arrayexpress/) (2), TISCH (http://tisch.comp-genomics.org/home/) (3) and published studies (Supplementary Figure S1). Datasets with more than 100 cells were retained in CellTracer. For each dataset, corresponding meta-data information including sample ID, organ/tissue origin, clinical treatment, biosample groups, primary/metastatic sites, and cell types, etc. were also integrated into CellTracer. Gene annotation files were collected from GENCODE database (https://www.gencodegenes.org/, release 41, GRCh38) (4) to identify different types of genes such as protein-coding genes, long non-coding RNAs (lncRNAs), pseudogenes, etc. We excluded cells that expressed fewer than 1,000 genes. Genes with a detectable expression in at least 1% of cells were retained. In this step, quality control (QC) and basic analysis (normalization, etc.) of these data were performed by R package Seurat (5). After raw data QC and normalization, CellTracer documented a number of 1,941,552 cells from 222 datasets (including 118 datasets of 42 diseases and 104 datasets of 80 normal organs and tissues).

Cellular clustering analysis

We performed unsupervised descending and clustering analysis of gene expression profile using the R package Seurat (v4.0.2, https://satijalab.org/seurat/) (5). After performing principal component analysis, a substantial subset of PCs was chosen for further classification using Seurat's JackStraw and ElbowPlot functions. The FindClusters, RunTSNE and RunUMAP

functions were used to conduct cell clustering and visualization, respectively. The resolution parameter of FindClusters function is adjusted from 0.1 to 0.9 (with an interval of 0.1) to provide clustering results at various resolutions, with higher resolution values corresponding to more cell clusters.

Cell type annotation

CellTracer performed cell type annotation by the following two strategies: (i) Using original cell-type annotation if provided by the original data source; (ii) Performing CELLiD method described by DISCO to annotate different cell types (6). In this step, the cell type of each cluster was determined using reference cell type marker genes and R codes of CELLiD method (https://github.com/JinmiaoChenLab/DISCO_manuscript/blob/master/CELLiD.R). The cell marker annotations were collected and combined from DISCO (6) and CellMarker (7). DISCO is a database of deeply integrated scRNA-seq data covering 107 tissues/cellines/organoids and 158 diseases. CellMarker is one of our previous works providing manually curated markers of diverse cell types. These databases collect cell marker annotations for both disease and healthy scRNA-seq data. To provide comprehensive annotations of diverse cell types, we integrated cell markers from both DISCO and CellMarker databases as cell type annotation reference. For each cell cluster, we used the comprehensive cell type annotation reference (such as normal cells, malignant cells, and disease cells) as input for CELLiD.

Cell development trajectories construction

The Monocle 2 package (v2.18.0) (8) was used to calculate pseudotime, states and further construct cell development trajectories. Monocle 2 works well with both relative expression

data and count-based measures. In general, it works best with transcript count data (http://cole-trapnell-lab.github.io/monocle-release/docs/). In our work, we use the gene counts as input matrix to Monocle 2. To work with count data, we set the expressionFamily parameter as negbinomial.size() to specify the negative binomial distribution. The project for Monocle 2 undertook Seurat's processing results containing quality filtered cells and the metadata of cells. Genes with an average expression greater than 0.1 were used for principal component analysis (PCA) and top 20 principal components were used for cell clustering. Further, featured genes were screened (q-value < 0.01) for cell sorting based on differentially expressed genes of clusters or cell types. To visualize trajectories in 2D space, the DDRTree algorithm was used to reduce the dimension. Further, the trajectory analyses results of Monocle 3 (v1.2.9) (9) were also included in CellTracer. Compared with Monocle 2, Monocle 3 changed the dimensional reduction method DDRTree to UMAP, which can better reflect data of high-dimensional space. The most important difference between Monocle 2 and 3 is that DDRTree based method assumes trajectories are connected into a single tree-like structure. While in Monocle3, multiple, disjoint graphs could be learned. Likewise, the top 20 principal components calculated by PCA were used for cell clustering and featured genes (q-value < 0.01) were selected for cell ordering. Finally, the pseudotime trajectory was visualized in the 2D space of UMAP. For each dataset, CellTracer provided trajectory analyses using Monocle 2, and Monocle 3 for different cell types, such as malignant cells, immune cells, stromal cells, etc.

Functional annotation data collection

In order to dissect the functional activation status and state transition of individual cellular populations, CellTracer collected functional gene sets including Gene Ontology (GO) (10),

biological pathways (11), hallmarks (12) and cellular states (13). The gene set variation analysis (GSVA) method (14) was used to evaluate the cellular functional activation status and states in each dataset. For annotation of GO, CellTracer collected 7,658 biological process (BP) gene sets, 1,738 molecular functions (MF) gene sets and 1,006 cellular component (CC) gene sets. For pathway annotation, a total of 2,982 biological pathway gene sets of Kyoto Encyclopedia of Genes and Genomes (KEGG), BioCarta, Reactome, and other biological pathway databases were collected. CellTracer also collected a number of 50 hallmark gene sets representing specific well-defined biological processes. The datasets of GO, biological pathways and hallmarks were downloaded from MSigDB (https://www.gsea-msigdb.org/gsea/msigdb/index.jsp) (11). To distinguish the functional states of different cancer cells, we downloaded the characteristic gene sets corresponding to the 14 functional states (such as stemness, invasion, metastasis, proliferation, EMT, angiogenesis, apoptosis, cell cycle, differentiation, DNA damage, DNA repair, hypoxia, inflammation, and quiescence) from our previous work (http://biocc.hrbmu.edu.cn/CancerSEA) (13).

Database construction

CellTracer can be freely visited at http://bio-bigdata.hrbmu.edu.cn/CellTracer/. The online web server of CellTracer was constructed by Java Server Pages language and deployed on the Tomcat software (v6, https://tomcat.apache.org/). The web pages were created by Hyper Text Markup Language (HTML) and controlled by JAVA programs (https://www.oracle.com/java/). All datasets of CellTracer were documented and managed based on the MySQL data source server (v5.5, http://www.mysql.com). Several Java script packages were implemented for result data creation and multi-level data cross-talk visualization. The jQuery (v1.11.3,

https://code.jquery.com) package was used to control the style of web pages. The Datatable (1.10.10, http://www.datatables.club/) package was used to generate data result table. The ECharts (V4.0, https://echarts.apache.org/) package was used to perform data visualization. All data processes and statistical analyses were performed using the R software (V4.2.1, https://cloud.r-project.org/).

References of Supplementary Methods

1. Barrett, T., Wilhite, S.E., Ledoux, P., Evangelista, C., Kim, I.F., Tomashevsky, M., Marshall, K.A., Phillippy, K.H., Sherman, P.M., Holko, M. *et al.* (2013) NCBI GEO: archive for functional genomics data sets--update. *Nucleic Acids Res*, **41**, D991-995.
2. Athar, A., Fullgrabe, A., George, N., Iqbal, H., Huerta, L., Ali, A., Snow, C., Fonseca, N.A., Petryszak, R., Papatheodorou, I. *et al.* (2019) ArrayExpress update - from bulk to single-cell expression data. *Nucleic Acids Res*, **47**, D711-D715.
3. Sun, D., Wang, J., Han, Y., Dong, X., Ge, J., Zheng, R., Shi, X., Wang, B., Li, Z., Ren, P. *et al.* (2021) TISCH: a comprehensive web resource enabling interactive single-cell transcriptome visualization of tumor microenvironment. *Nucleic Acids Res*, **49**, D1420-D1430.
4. Frankish, A., Diekhans, M., Jungreis, I., Lagarde, J., Loveland, J.E., Mudge, J.M., Sisu, C., Wright, J.C., Armstrong, J., Barnes, I. *et al.* (2021) Gencode 2021. *Nucleic Acids Res*, **49**, D916-D923.
5. Satija, R., Farrell, J.A., Gennert, D., Schier, A.F. and Regev, A. (2015) Spatial reconstruction of single-cell gene expression data. *Nat Biotechnol*, **33**, 495-502.
6. Li, M., Zhang, X., Ang, K.S., Ling, J., Sethi, R., Lee, N.Y.S., Ginhoux, F. and Chen, J. (2022) DISCO: a database of Deeply Integrated human Single-Cell Omics data. *Nucleic Acids Res*, **50**, D596-D602.
7. Zhang, X., Lan, Y., Xu, J., Quan, F., Zhao, E., Deng, C., Luo, T., Xu, L., Liao, G., Yan, M. *et al.* (2019) CellMarker: a manually curated resource of cell markers in human and mouse. *Nucleic Acids Res*, **47**, D721-D728.
8. Qiu, X., Mao, Q., Tang, Y., Wang, L., Chawla, R., Pliner, H.A. and Trapnell, C. (2017) Reversed graph embedding resolves complex single-cell trajectories. *Nat Methods*, **14**, 979-982.
9. Cao, J., Spielmann, M., Qiu, X., Huang, X., Ibrahim, D.M., Hill, A.J., Zhang, F., Mundlos, S., Christiansen, L., Steemers, F.J. *et al.* (2019) The single-cell transcriptional landscape of mammalian organogenesis. *Nature*, **566**, 496-502.
10. Gene Ontology, C. (2021) The Gene Ontology resource: enriching a GOld mine. *Nucleic Acids Res*, **49**, D325-D334.
11. Liberzon, A., Birger, C., Thorvaldsdottir, H., Ghandi, M., Mesirov, J.P. and Tamayo, P. (2015) The Molecular Signatures Database (MSigDB) hallmark gene set collection. *Cell systems*, **1**, 417-425.
12. Hanahan, D. and Weinberg, R.A. (2011) Hallmarks of cancer: the next generation. *Cell*, **144**, 646-674.
13. Yuan, H., Yan, M., Zhang, G., Liu, W., Deng, C., Liao, G., Xu, L., Luo, T., Yan, H., Long, Z. *et al.* (2019) CancerSEA: a cancer single-cell state atlas. *Nucleic Acids Res*, **47**, D900-D908.

14. Hänzelmann, S., Castelo, R. and Guinney, J. (2013) GSVA: gene set variation analysis for microarray and RNA-seq data. *BMC bioinformatics*, **14**, 7.

Supplementary Figures

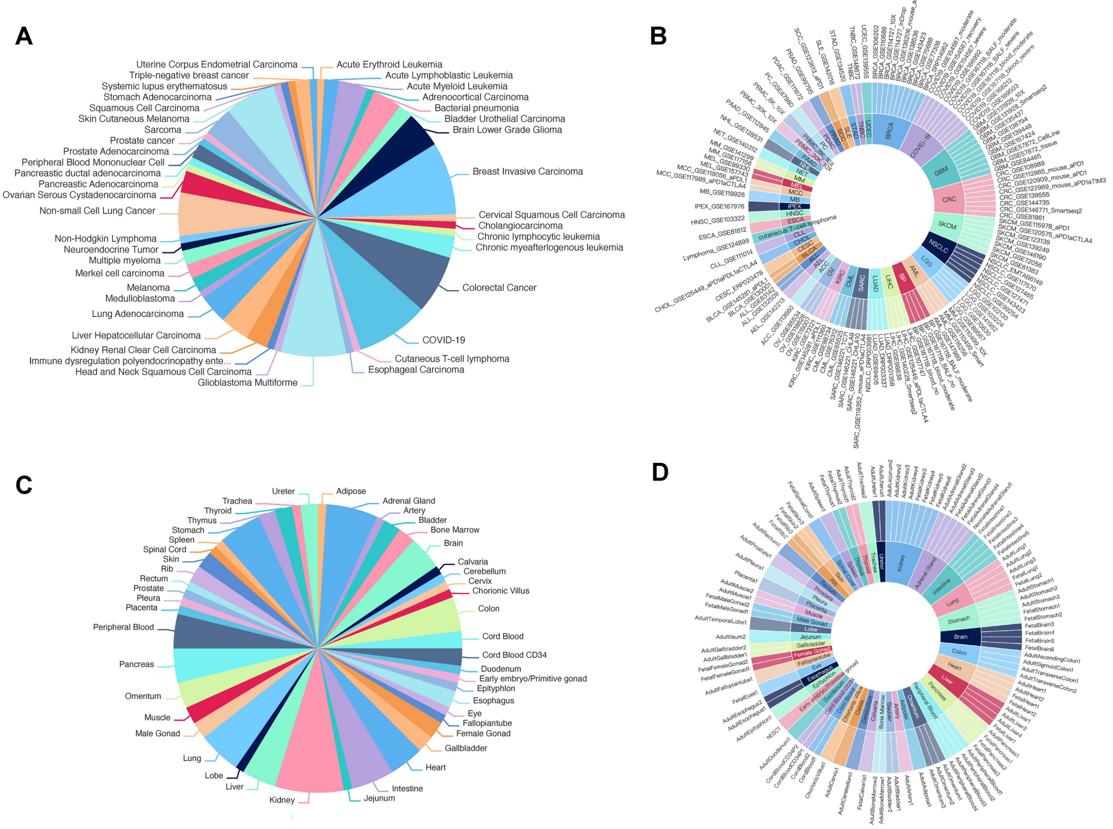

Figure S1. Overview of datasets in CellTracer. **(A-B)** The diseases distribution across 118 datasets. **(C-D)** The organs and tissues distribution across 104 healthy datasets.

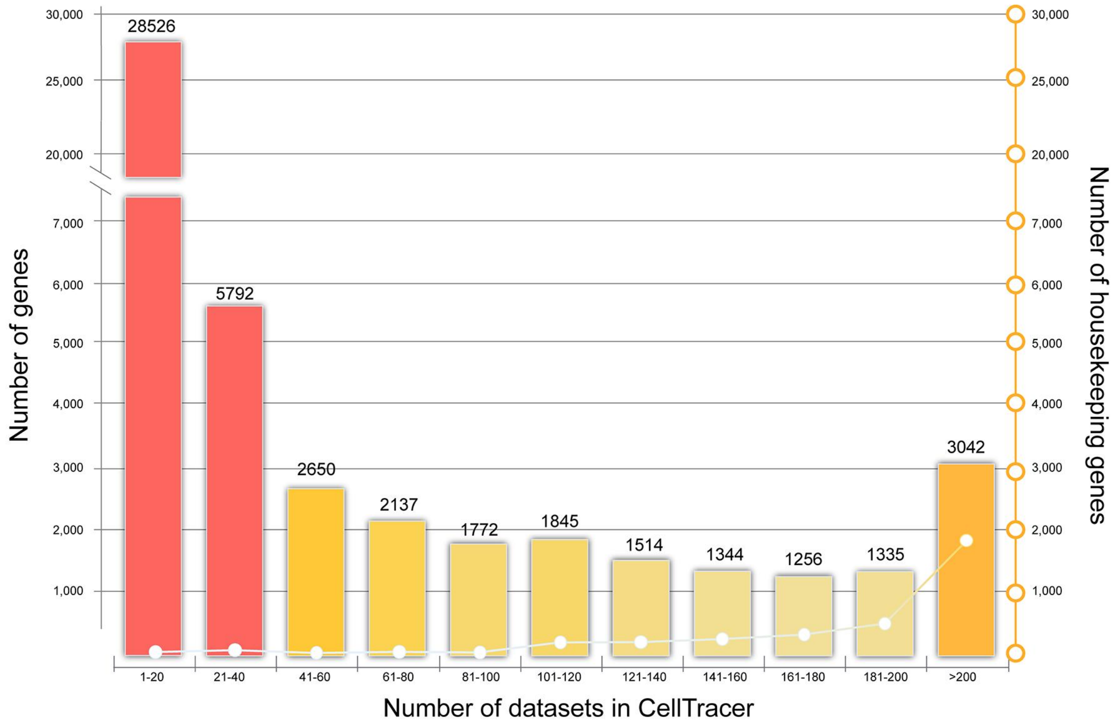

Figure S2. Overview of genes expressed in different datasets of CellTracer. The y axis in left indicates the number of genes. The y axis in right indicates the number of housekeeping genes in different groups. The x axis indicates breaks of different number of datasets.

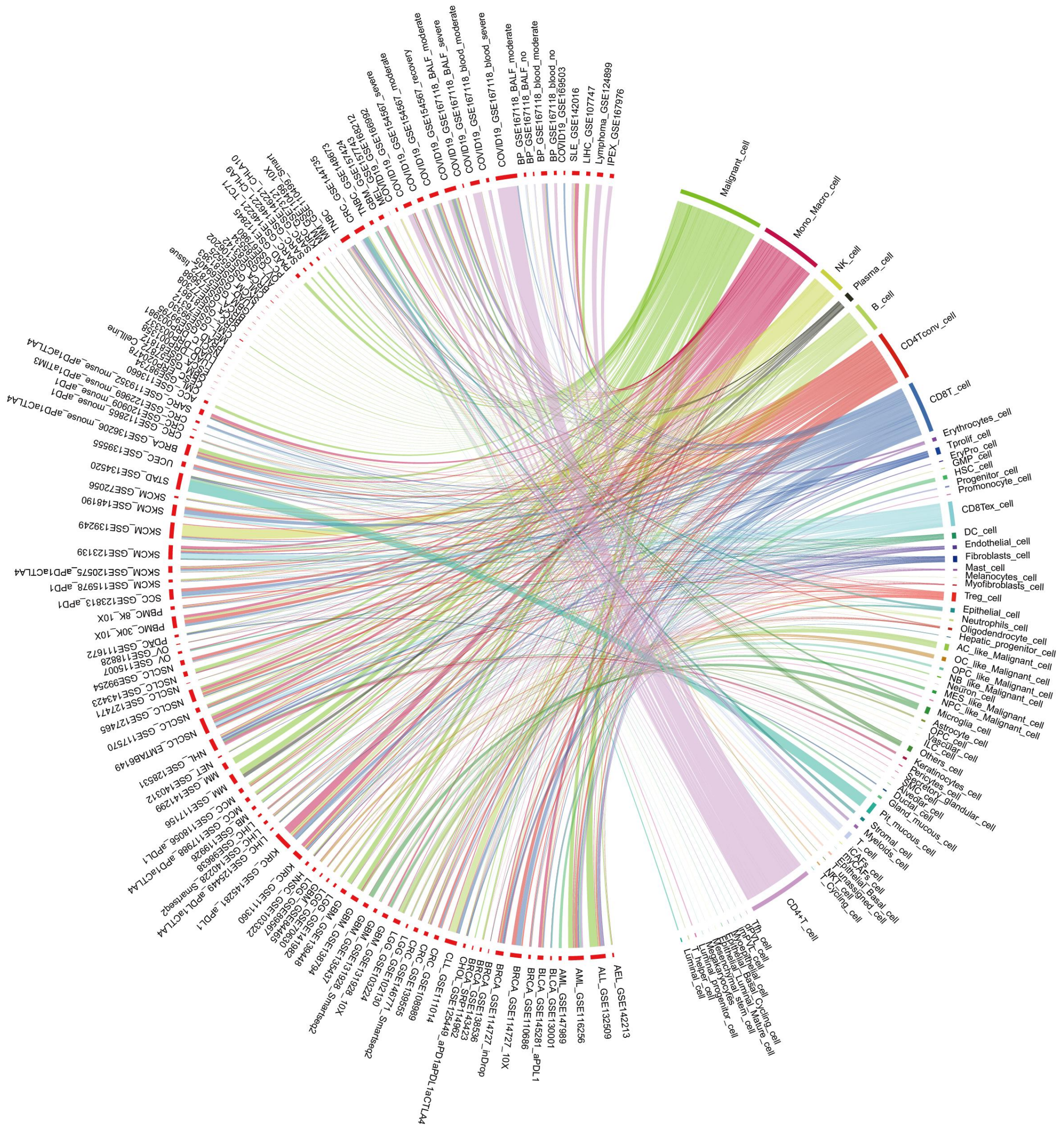

Figure S3. Cell types and their association with disease datasets in CellTracer.

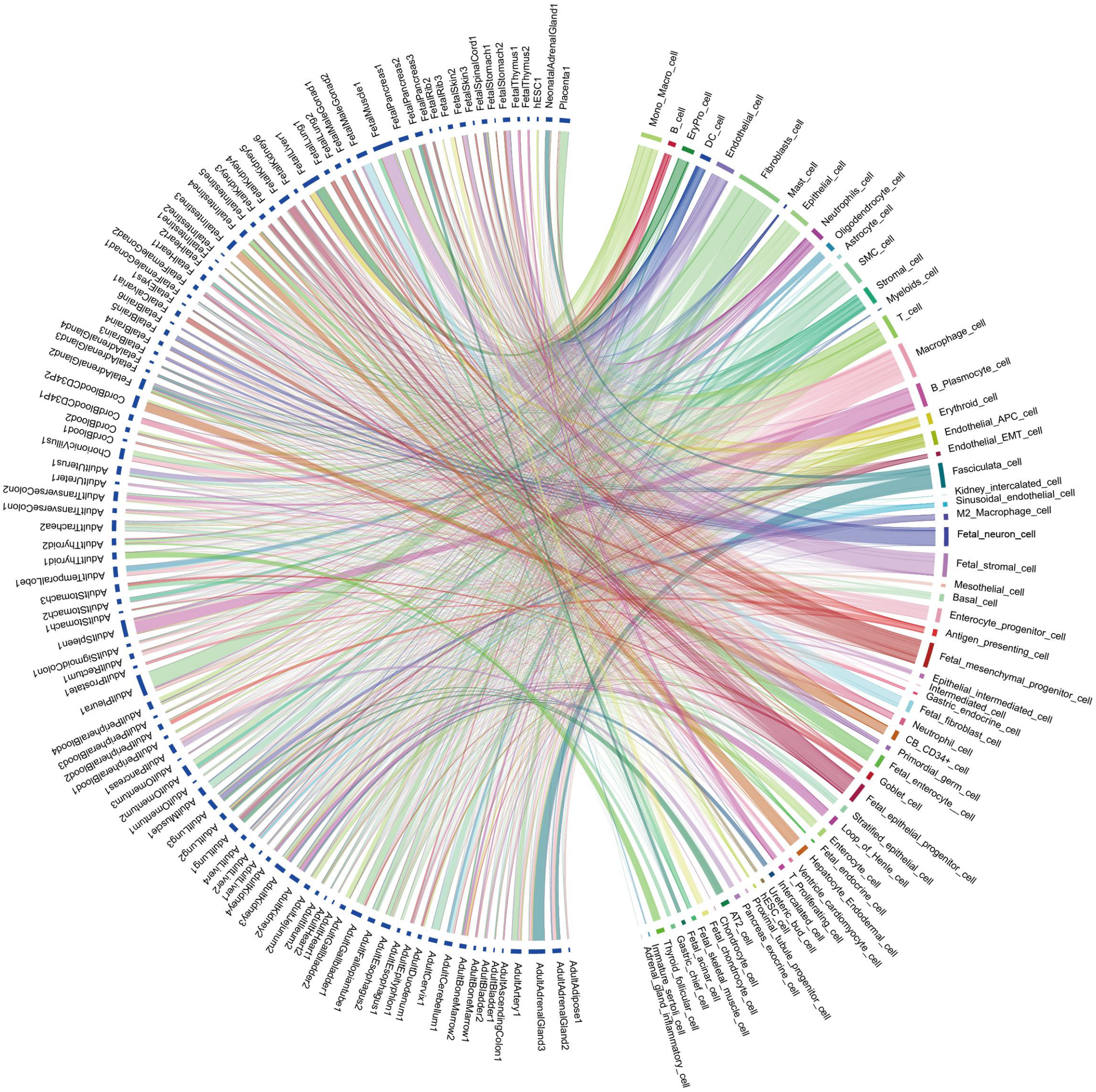

Figure S4. Cell types and their association with normal datasets in CellTracer.

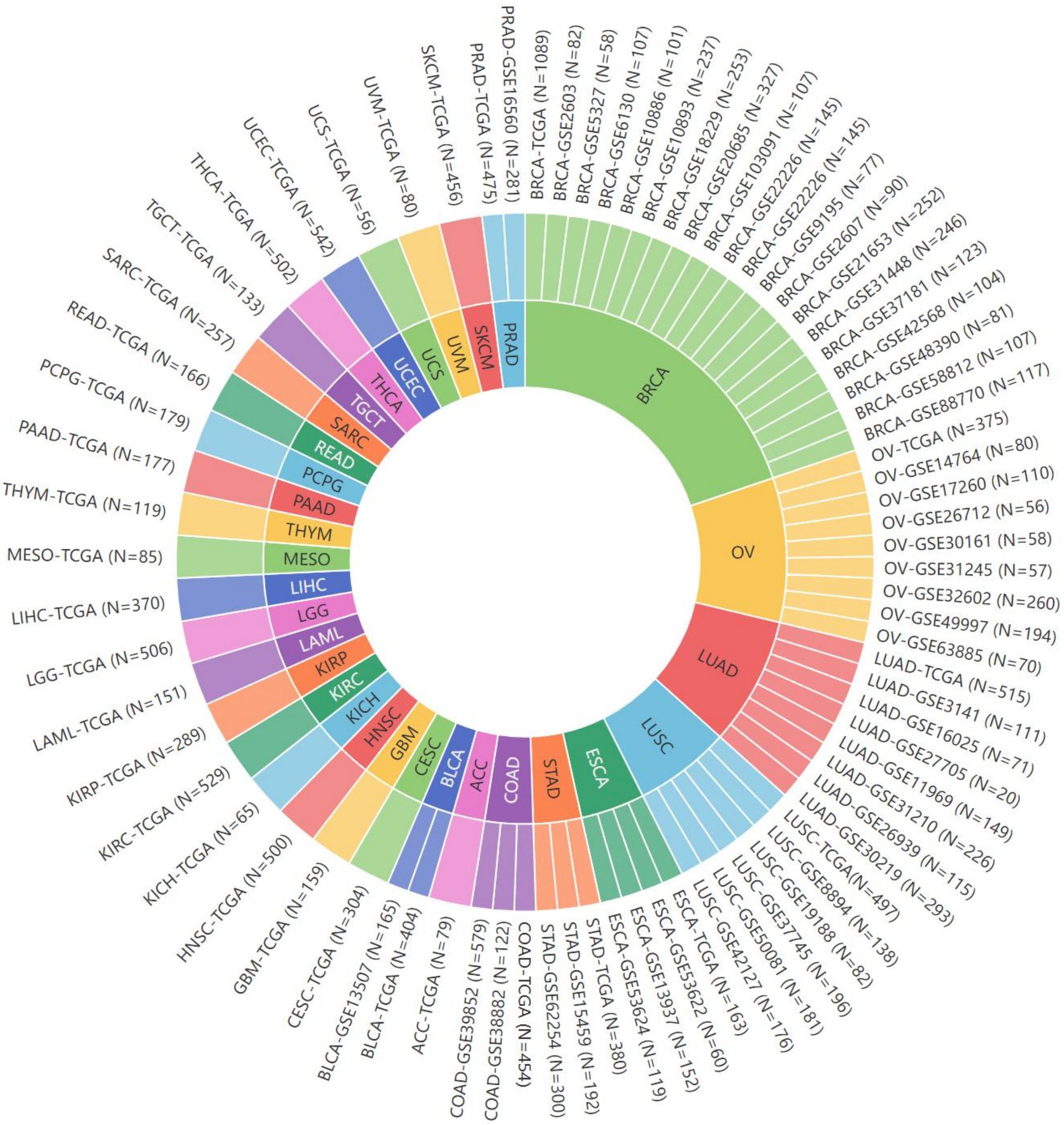

Figure S5. Overview of bulk expression datasets in CellTracer.

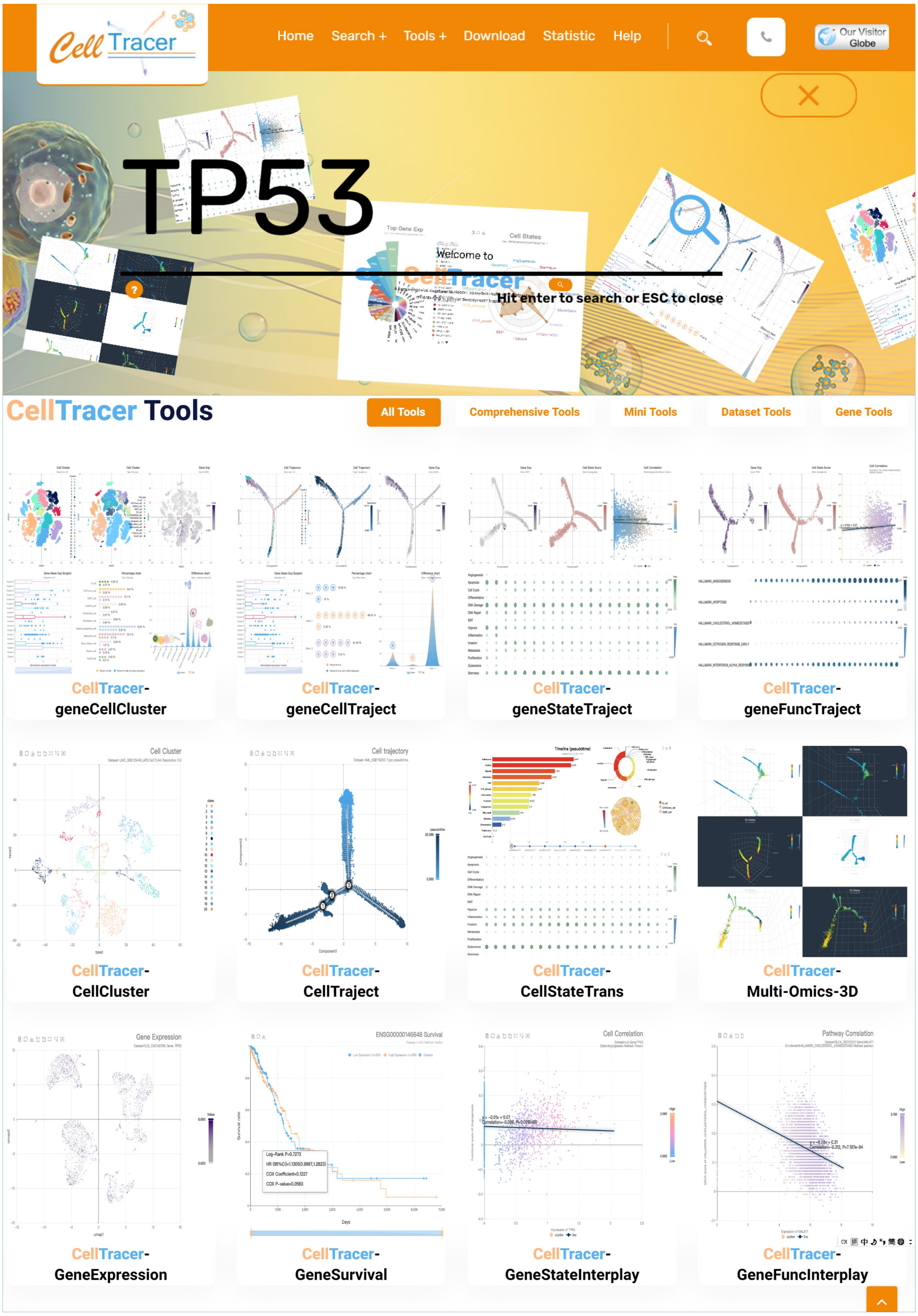

Figure S6. A screenshot of CellTracer 'Home' page. The top panel illustrates the quick search interface whereas the bottom illustrates a panel of easy-to-use tools.

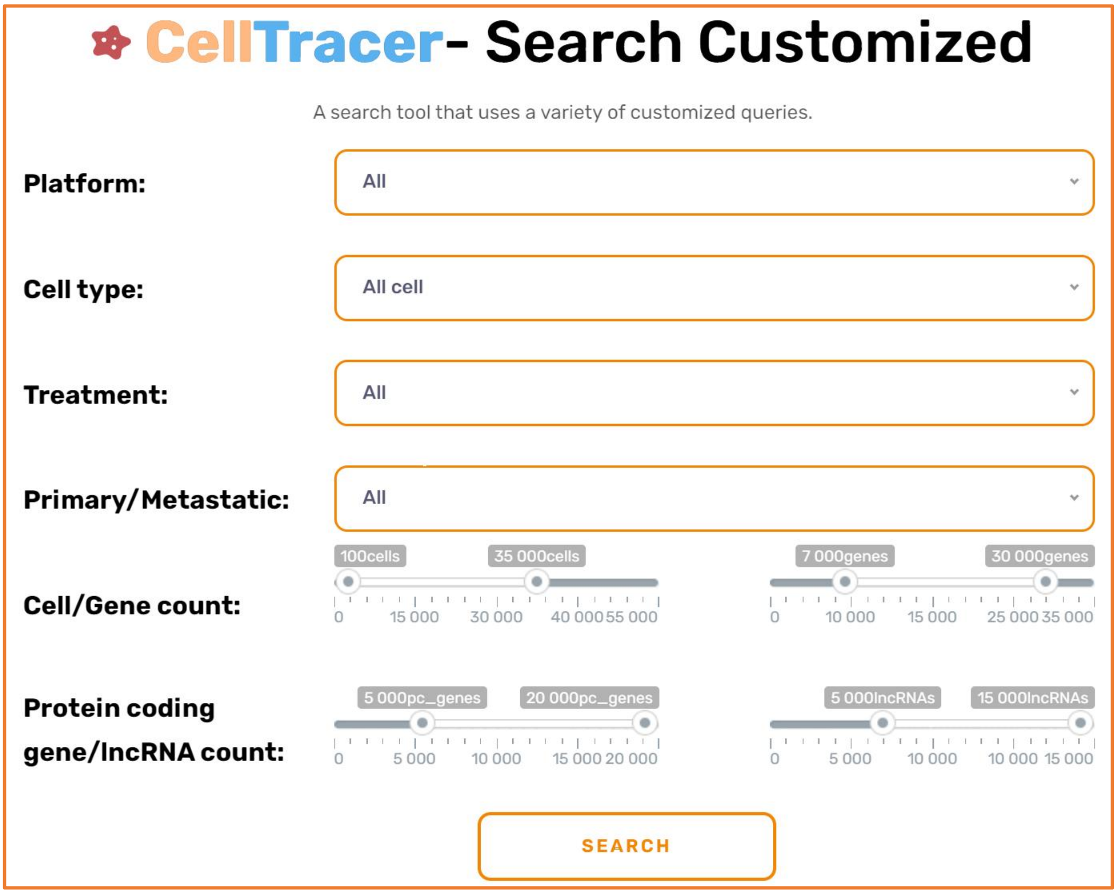

Figure S7. A screenshot of CellTracer 'Advanced search' page.

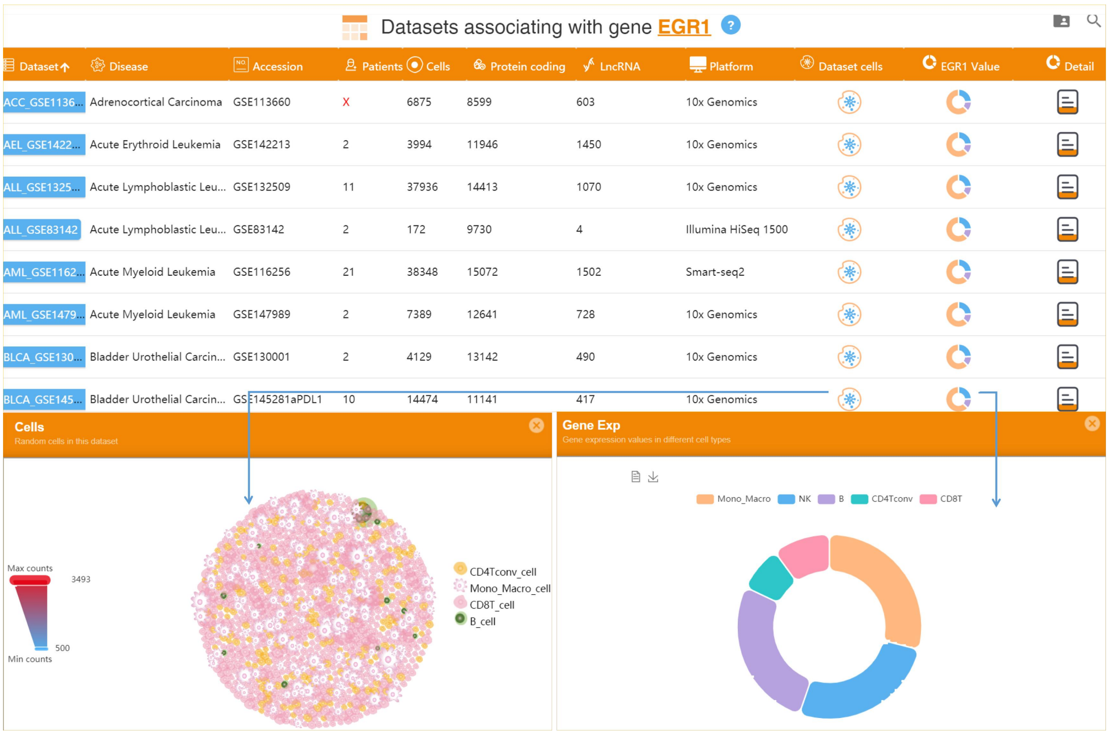

Figure S8. Illustration of cell types composition and distribution of gene expressions by CellTracer.

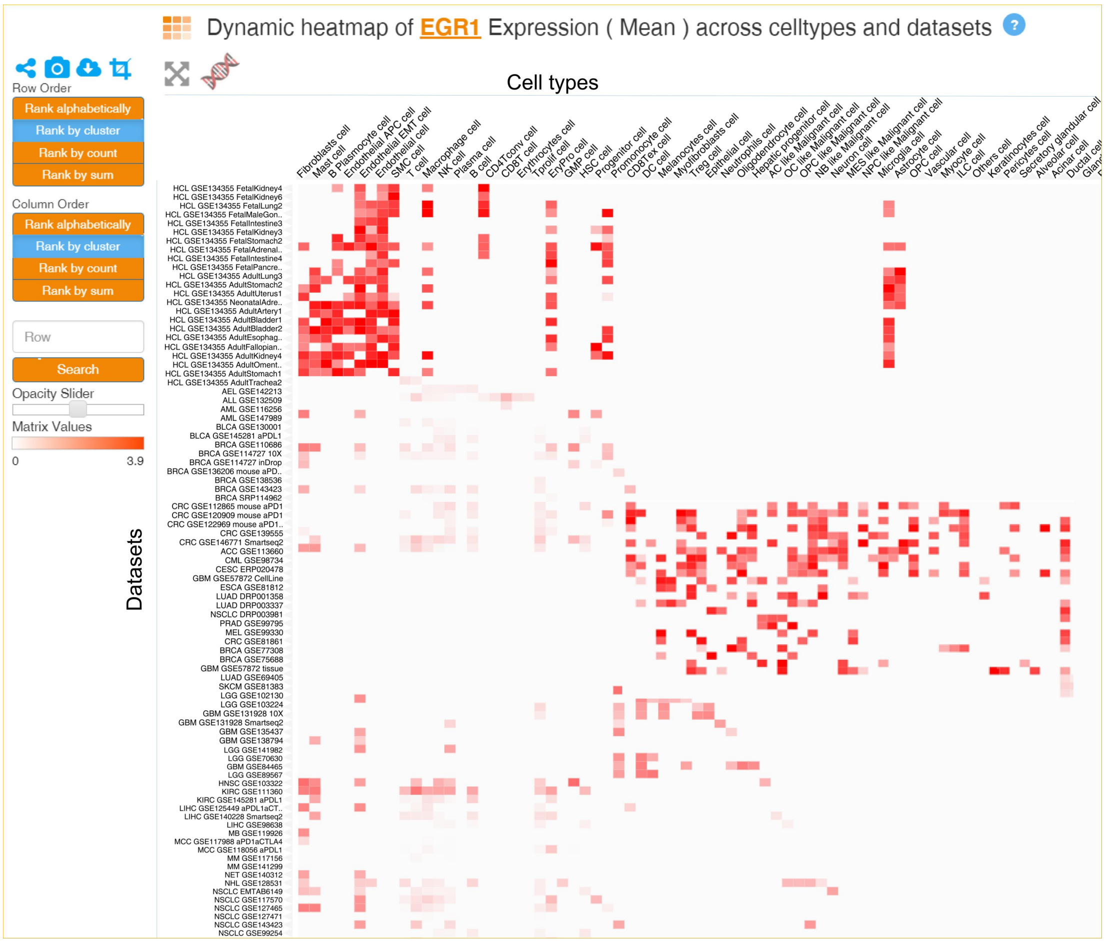

Figure S9. An interactive heatmap illustrating the detailed gene expression across different datasets and cell types. Users can directly reorder the rows and columns of the heatmap by setting different ranking parameters in the top left panel.

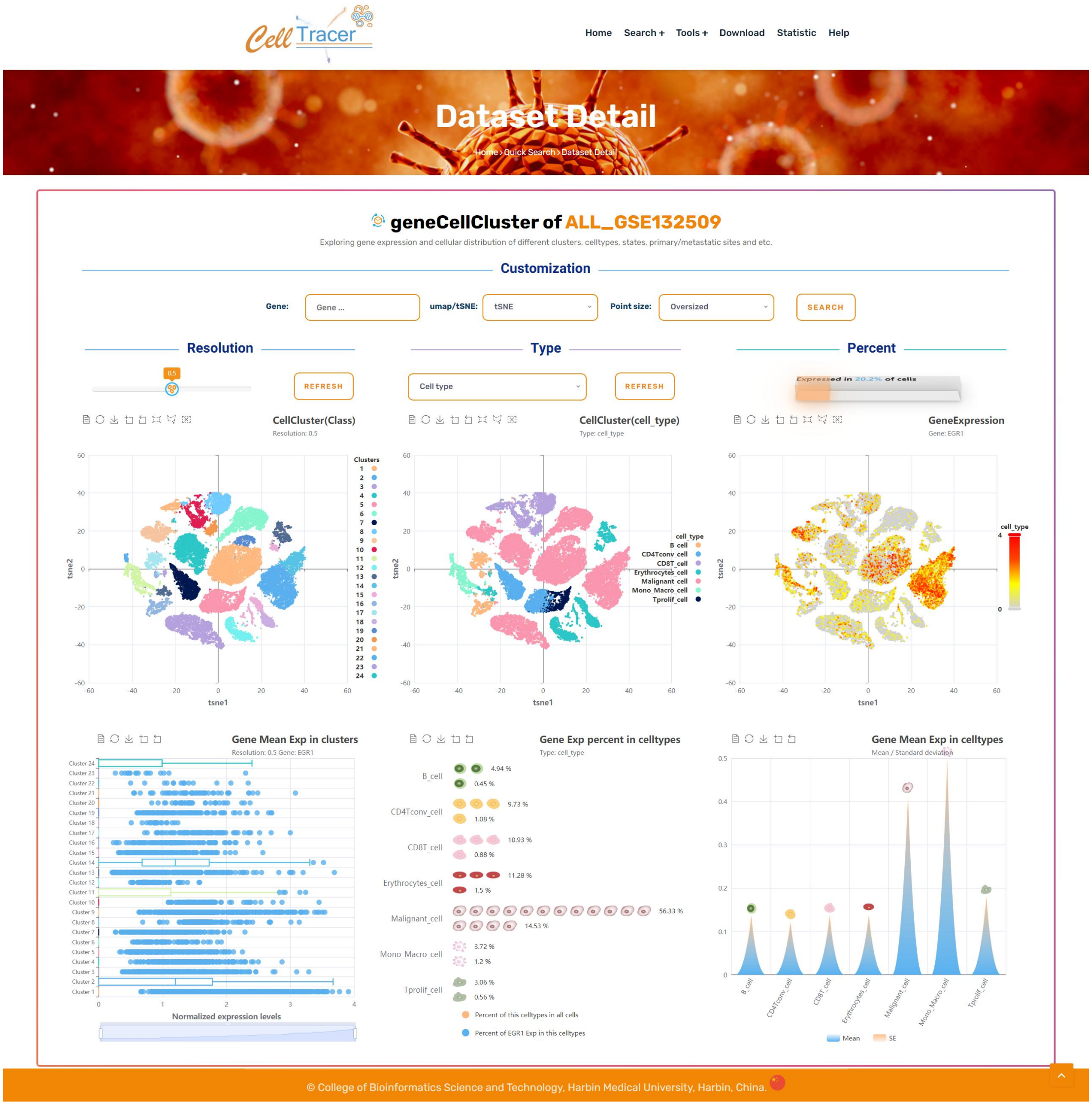

Figure S10. A screenshot of GeneCellCluster tool in CellTracer.

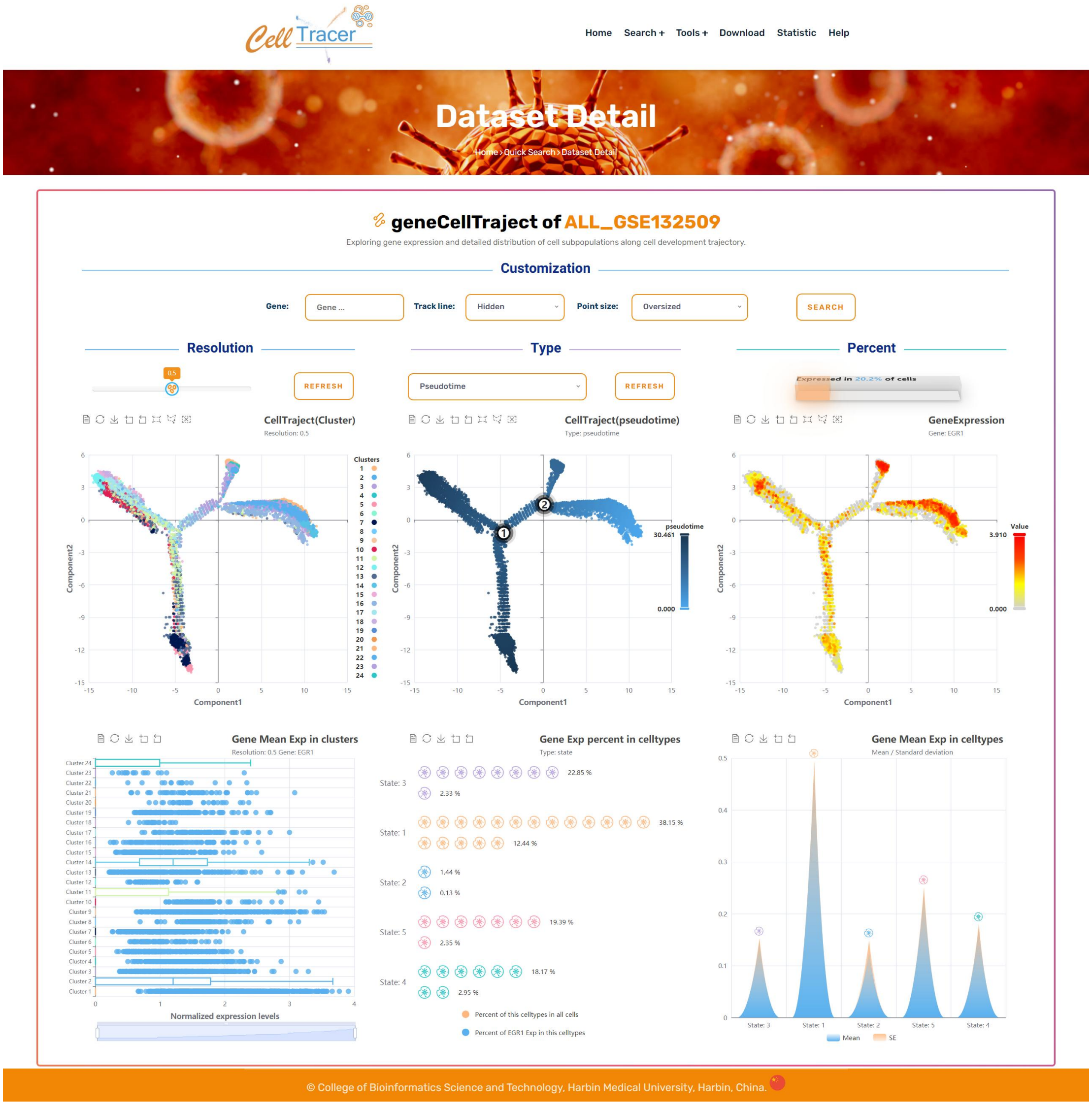

Figure S11. A screenshot of GeneCellTraject tool in CellTracer.

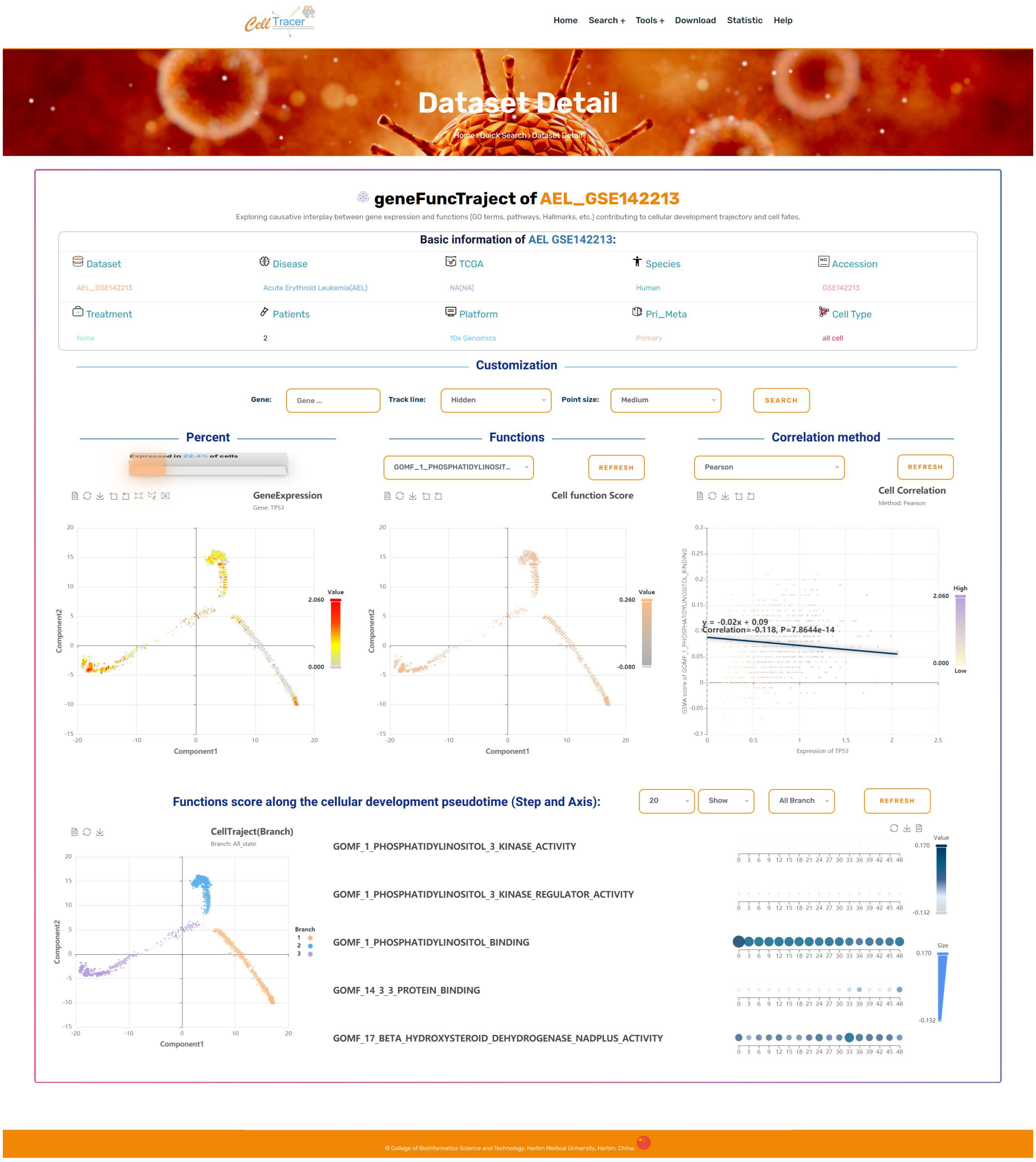

Figure S12. A screenshot of GeneFuncTraject tool in CellTracer. The top panel illustrates the distribution functional scores across different cells and performs correlation analysis between gene expression and functional contexts. The bottom panel illustrates the functional variation along developmental pseudotime.

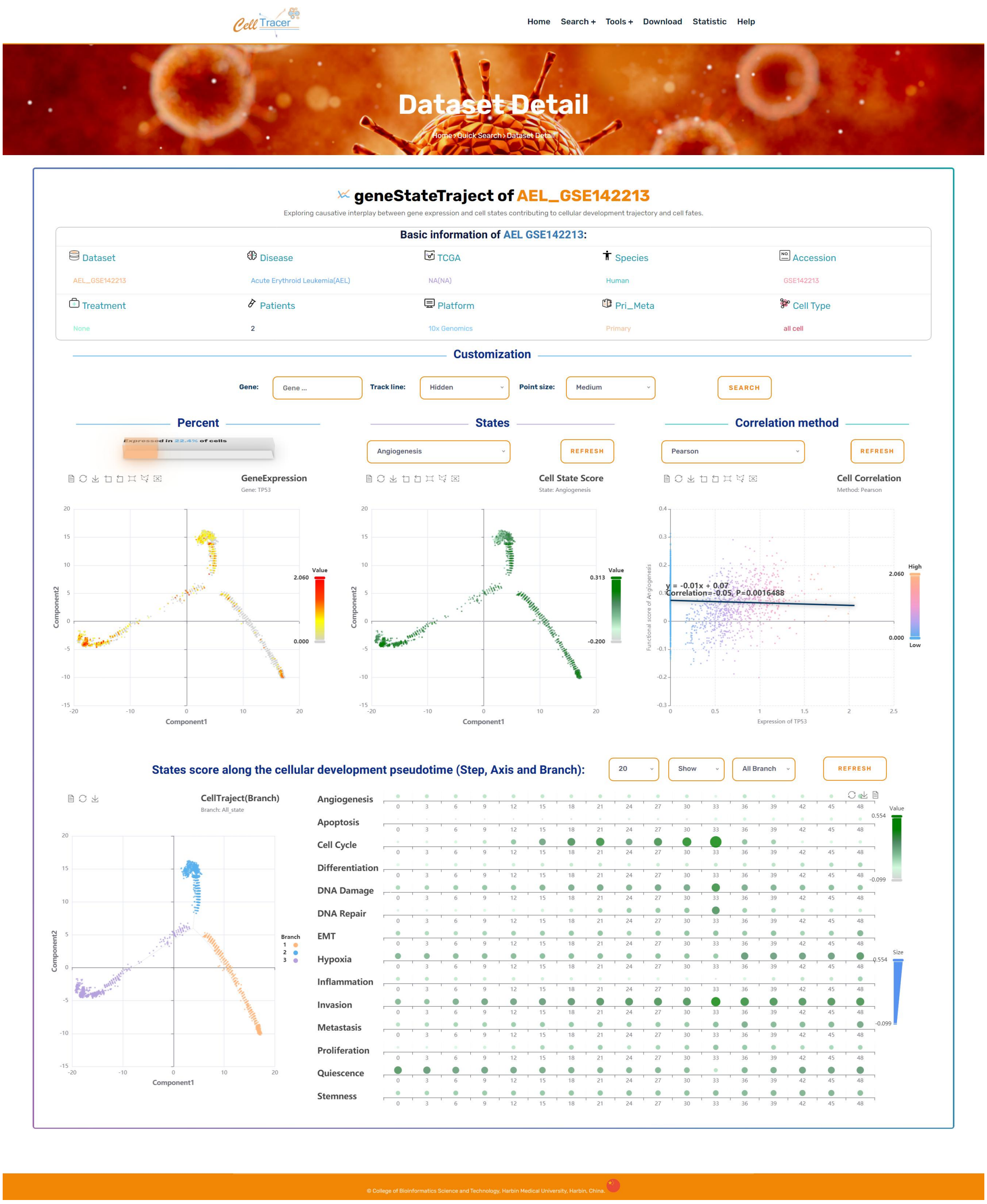

Figure S13. A screenshot of GeneStateTraject tool in CellTracer. The top panel illustrates the distribution of cellular states across different cells and performs correlation analysis between gene expression and cellular states. The bottom panel illustrates the states variation along developmental pseudotime.

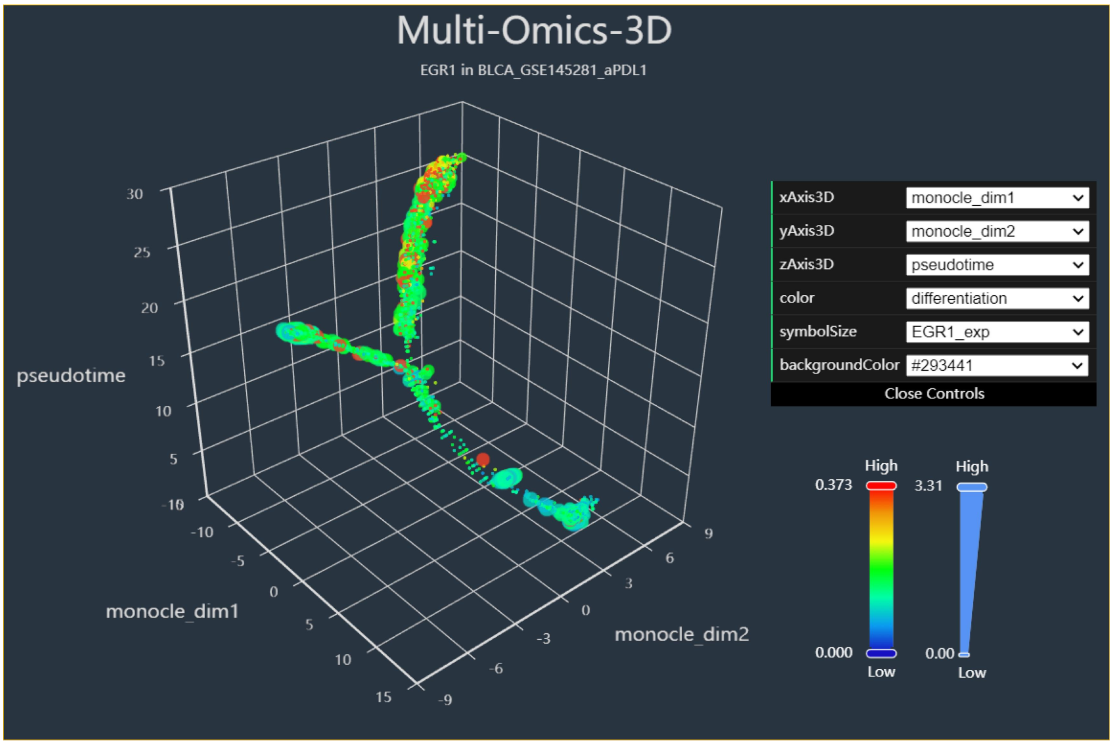

Figure S14. A screenshot of Multi-Omics-3D tool in CellTracer. Users can perform combination and visualization of multilevel features and their crosstalk by mapping these features to the x, y, z axes, node colours and symbol size.

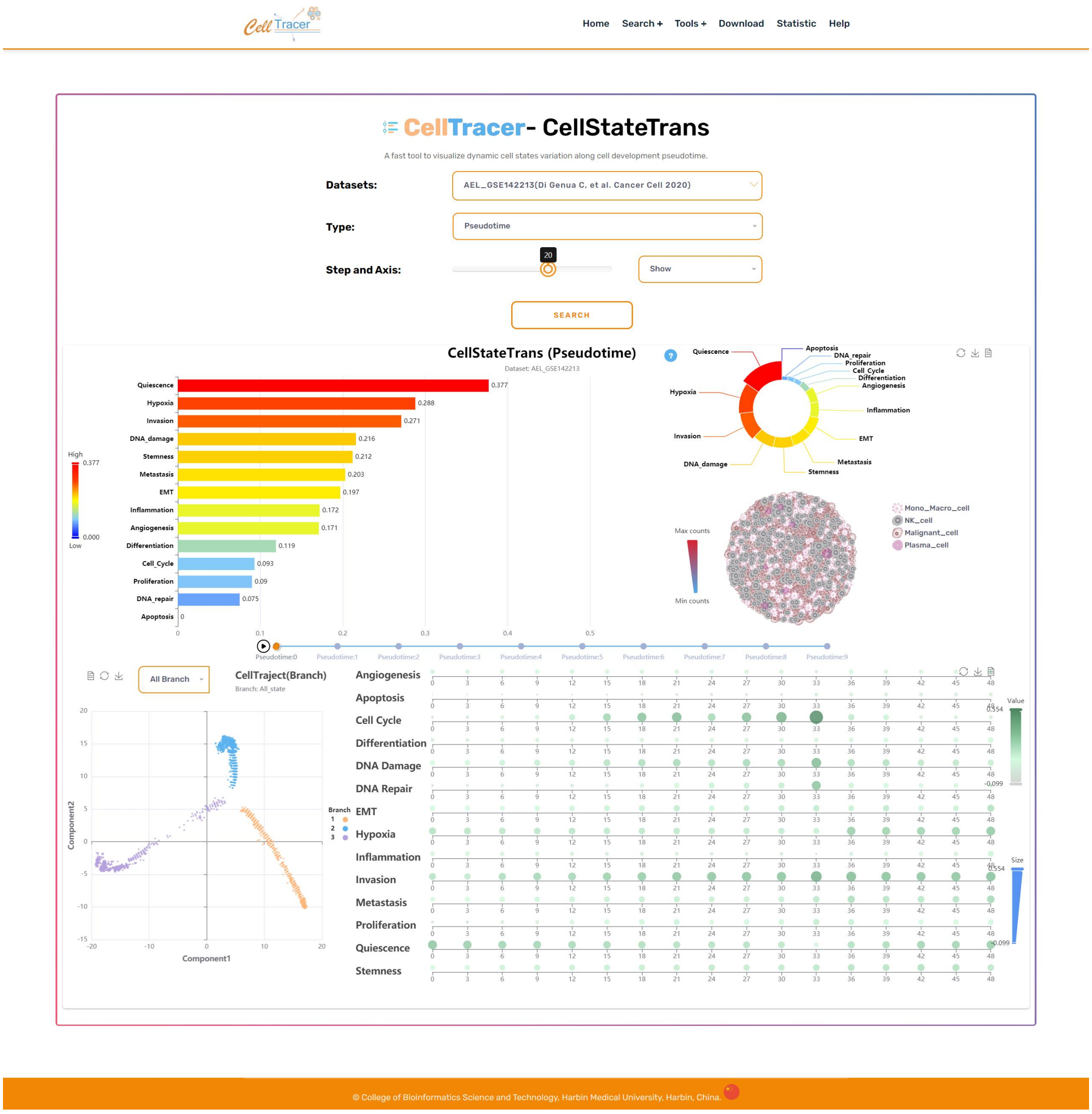

Figure S15. A screenshot of CellStateTrans tool in CellTracer. Users can study the dynamic change of cellular states/behaviours along continuous development pseudotime by manually control of time points.

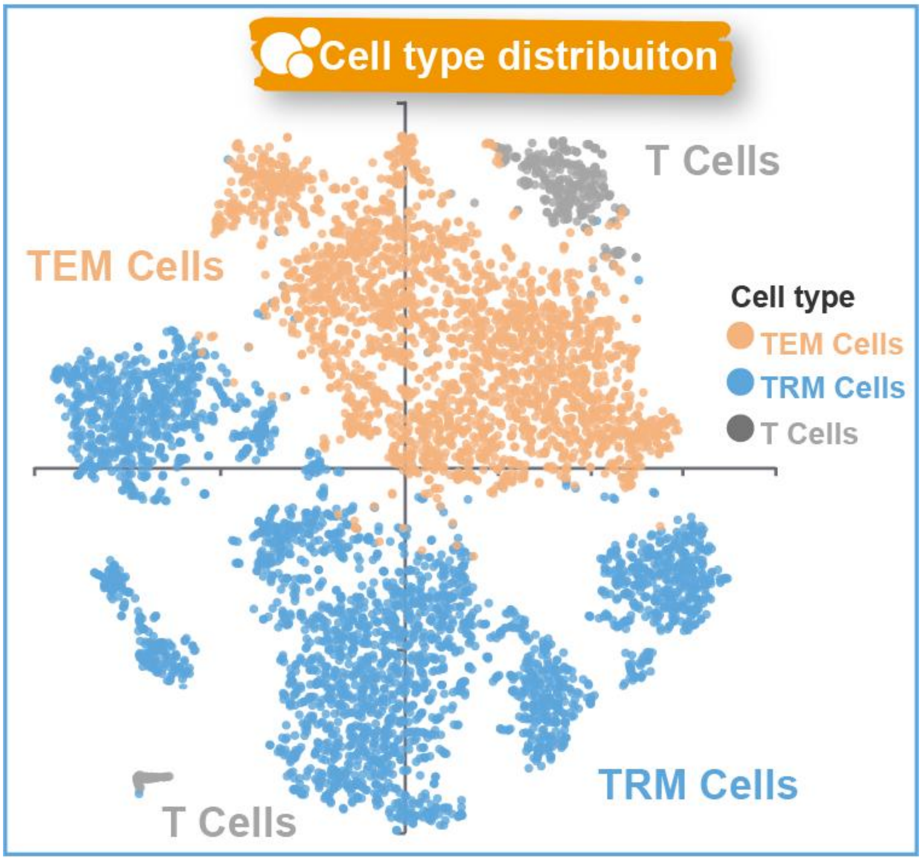

Figure S16. Cell type annotation of different clusters.

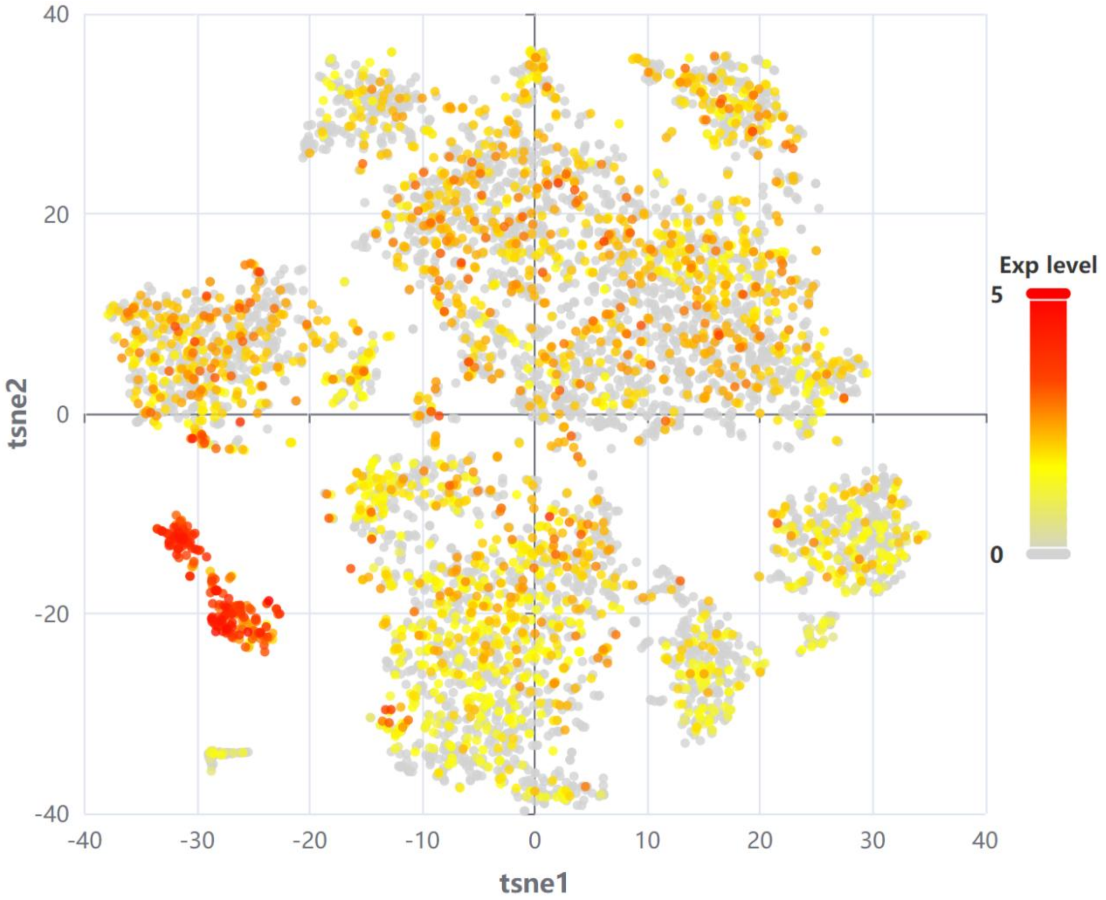

Figure S17. The expression distribution of HMGB2 in different cell clusters.

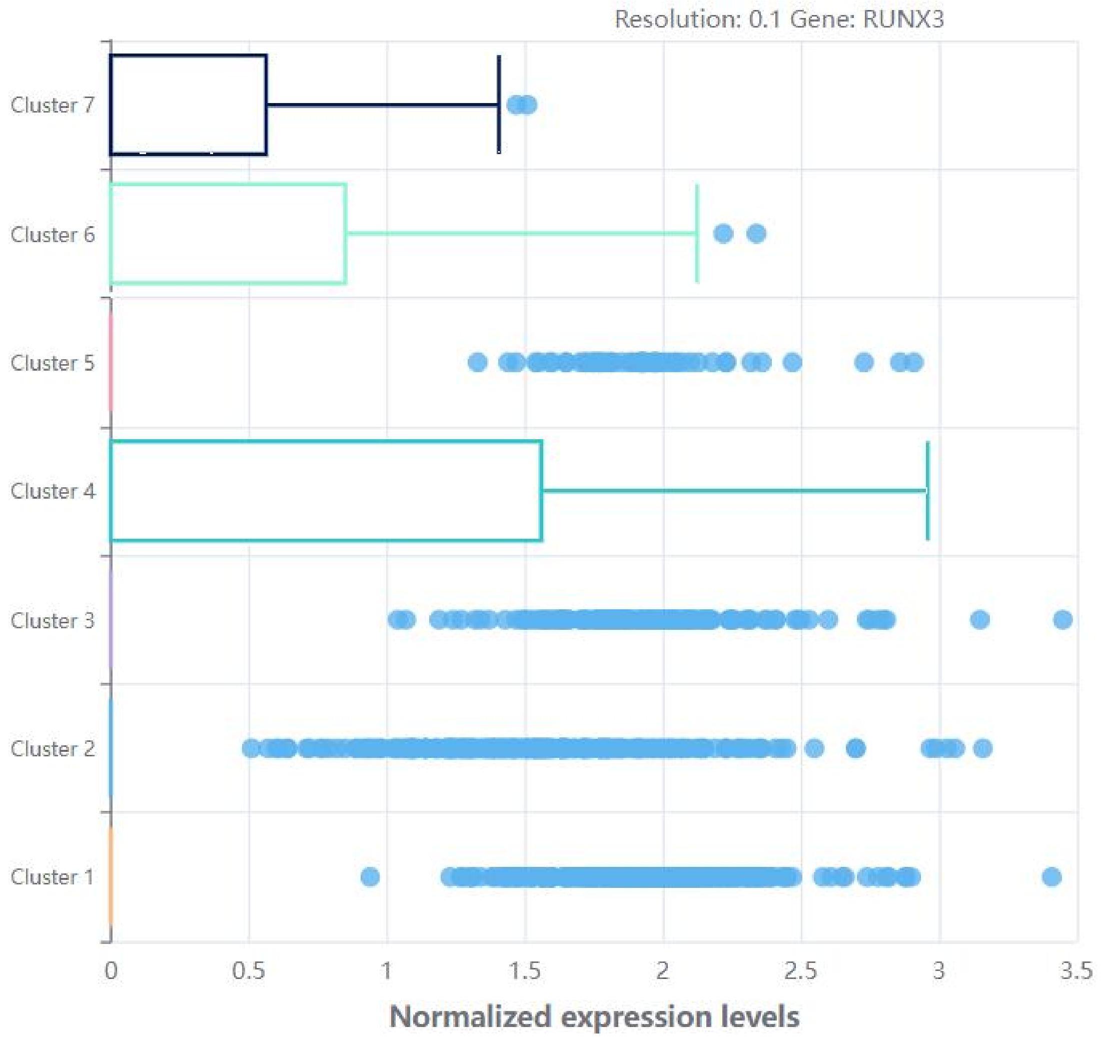

Figure S18. The expression distribution of RUNX3 in different cellular clusters. This figure was generated by GeneCellCluster tool of CellTracer.

.

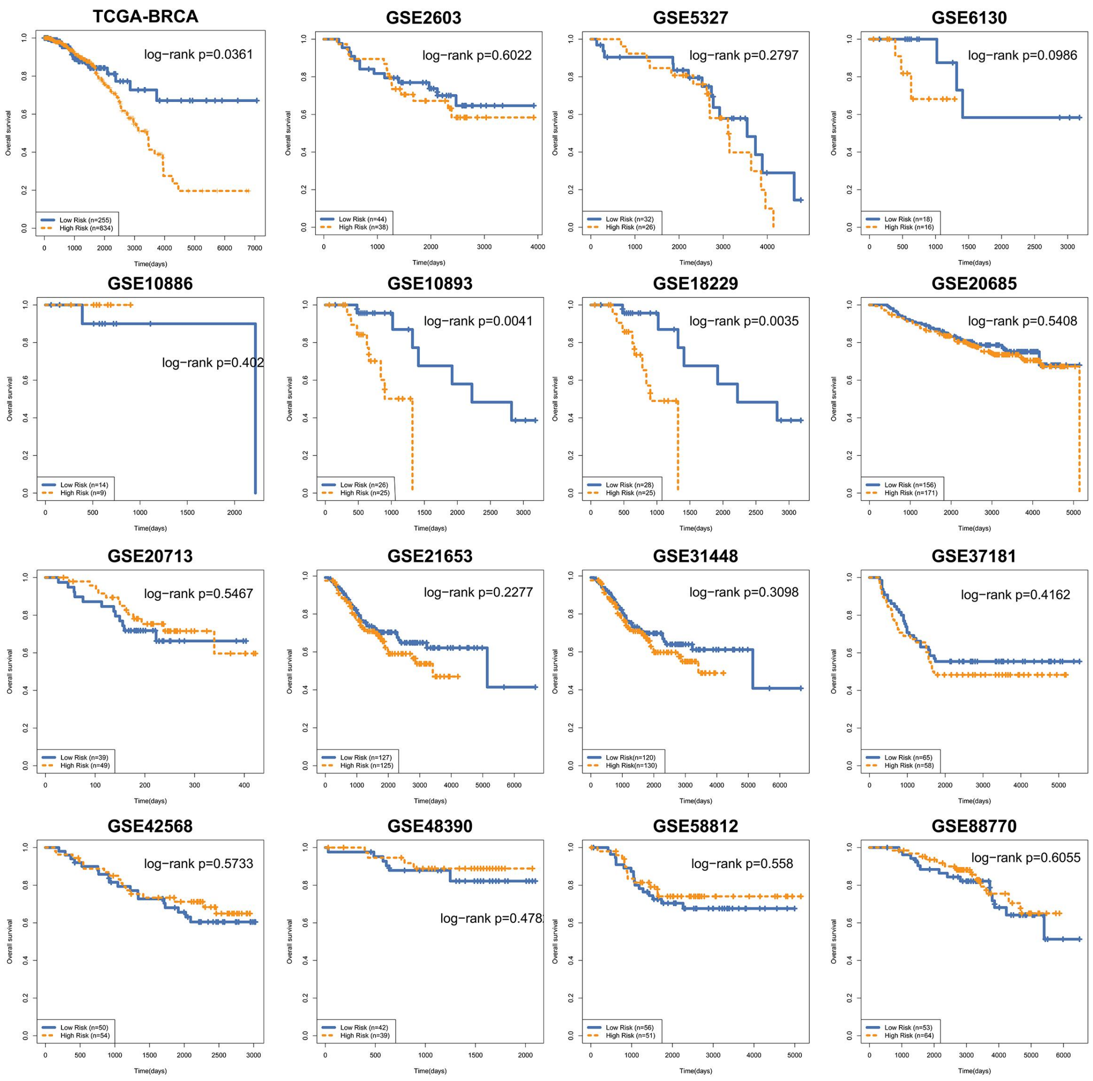

Figure S19. Survival analysis of ITGAE in a panel of 16 breast cancer bulk datasets based on GeneSurvival tool in CellTracer.

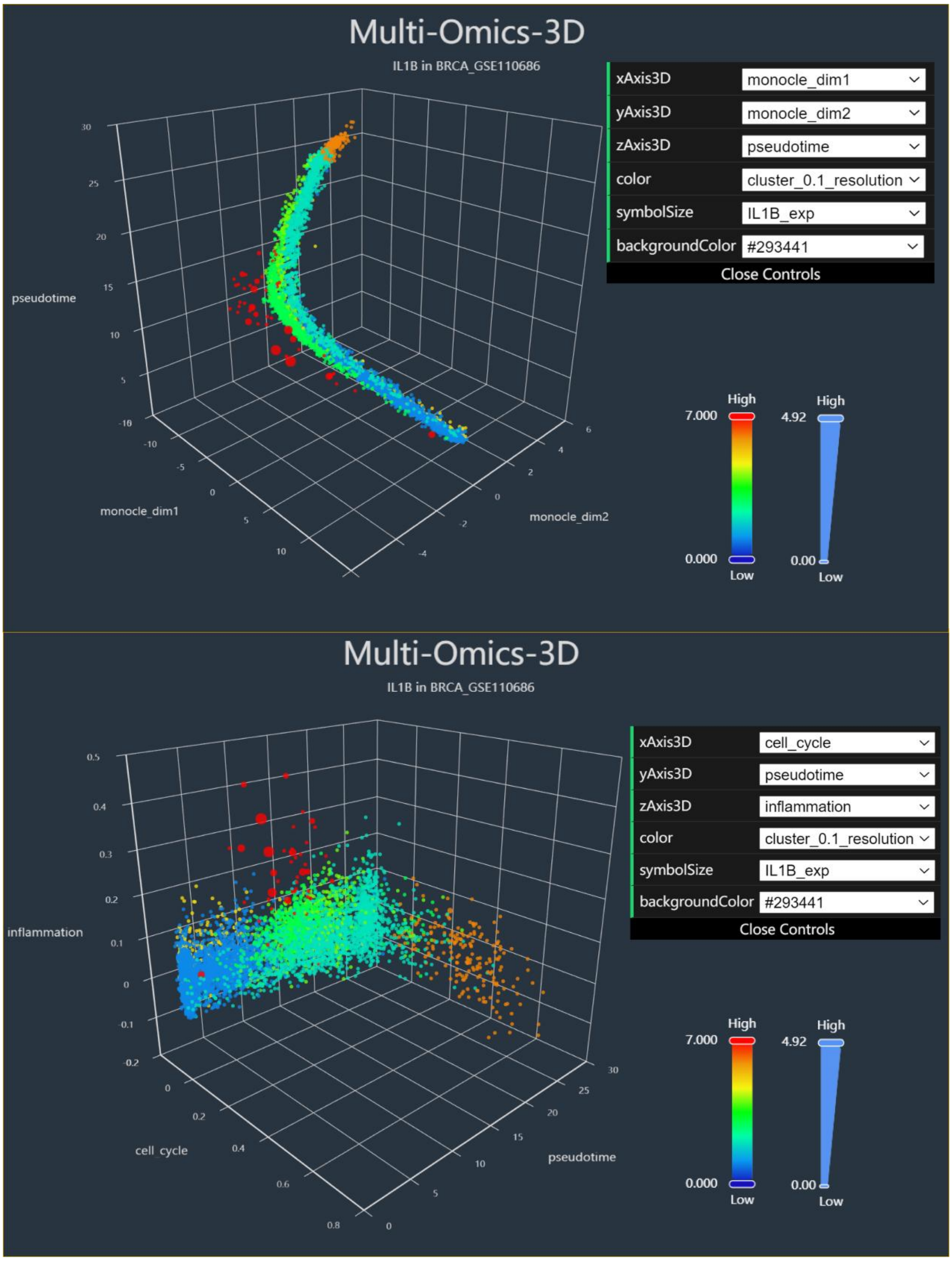

Figure S20. Examples of Multi-Omics-3D tool in characterizing cellular states of Monocyte/Macrophage cells (nodes in red colour). This cluster of cells exhibit high inflammation activities with increased expression of IL1B which is an important mediator of the inflammatory response.
